# Insight into the Li-Storage Property of Surface-Modified Ti_2_Nb_10_O_29_ Anode Material for High-Rate Application

**DOI:** 10.1021/acsami.3c14174

**Published:** 2023-11-16

**Authors:** Nikhitha Joseph, Haojie Fei, Constantin Bubulinca, Marek Jurca, Matej Micusik, Maria Omastova, Petr Saha

**Affiliations:** † Centre of polymer systems, Tomas Bata University in Zlín, 760 01 Zlín, Czech Republic; ‡ Polymer Institute, Slovak Academy of Sciences, Dúbravská cesta 9, 845 41 Bratislava, Slovakia; § University Institute, Tomas Bata University in Zlín, 760 01 Zlín, Czech Republic

**Keywords:** Intercalation anode, lithium-ion battery, titanium niobium oxide, carbon−copper coating, oxygen deficiency

## Abstract

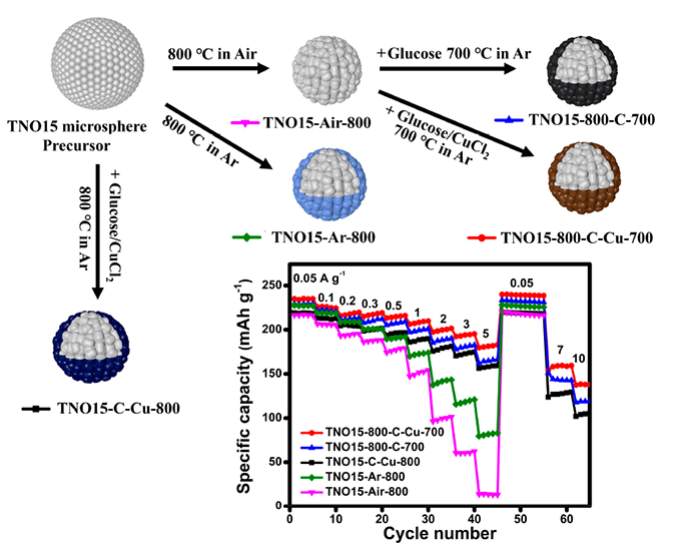

Ti-based anode materials are considered to be an alternative to graphite anodes to accomplish high-rate application requirements. Ti_2_Nb_10_O_29_ (TNO15) has attracted much attention due to its high lithium storage capacity through the utilization of multiple redox couples and a suitable operating voltage window of 1.0 to 2.0 V vs Li/Li^+^. However, poor intrinsic electronic conductivity has limited the futuristic applicability of this material to the battery anode. In this work, we report the modification of TNO15 by introducing oxygen vacancies and using few-layered carbon and copper coatings on the surface to improve its Li^+^ storage property. With the support of the galvanostatic intermittent titration technique (GITT), we found that the diffusion coefficient of carbon/copper coated TNO15 is 2 orders of magnitude higher than that of the uncoated sample. Here, highly conductive copper metal on the surface of the carbon-coated oxygen-vacancy-incorporated TNO15 increases the overall electronic and ionic conductivity. The prepared TNO15-800-C-Cu-700 half-cell shows a significant rate capability of 92% when there is a 10-fold increase in the current density. In addition, the interconnected TNO15 nanoparticles create a porous microsphere structure, which enables better Li-ion transportation during charge/discharge process, and experiences an enhancement after the carbon and copper coating on the surface of the primary TNO15 nanocrystallites.

## Introduction

Lithium-ion batteries (LIBs) are the key technology for the development of portable electronics and large-scale energy storage applications, especially for electric vehicles. Commercially available lithium-ion batteries utilize graphite anodes owing to their low cost, abundance, relatively large specific capacity of 379 mAh g^–1^, high energy density, and long cycle life.^1^ Nevertheless, graphite is limited to high-rate performances due to the formation of a lithium-permeable solid electrolyte interphase (SEI) on its surface, which increases the impedance of the anode. This formation consumes a large amount of lithium from the cathode irreversibly during initial cycling and causes large capacity losses over time. Besides, particle fracture and dendrite growth arise from its low working potential (0.1 V vs Li/Li^+^), close to that of the plating potential of lithium metal, resulting in increasing potential safety risks.^2^ Thus, low-voltage anode materials are limited for the high-rate requirements, and a small overpotential results in lithium plating on the surface of the electrode material, which will further result in an unsatisfactory safety level.^3,4^ Increasing efforts have been devoted to developing high-voltage anode materials, especially titanium- and niobium-based anodes like Li_4_Ti_5_O_12_ (LTO) and Nb_2_O_5_, which are better alternatives for the graphite anode.^5,6^ Owing to their excellent lithium insertion kinetics by utilizing Ti^4+^/Ti^3+^ and Nb^5+/^Nb^4+^ redox couples within a safe voltage range (>1.0 V vs Li/Li^+^), the lithium dendrite growth and the formation of the SEI layer are limited.^7−9^ The LTO-based cells commercialized by Toshiba in SCiB batteries provide a long life of over 20 000 cycles, excellent low-temperature performance, and a high level of safety. Nevertheless, these materials are limited by their low theoretical capacity, poor rate performance, and low power density associated with their low conductivity.^10^ Various strategies have been developed to solve these problems; for example, Yi et al. reported Cr (III) doping into spinel-type Li_4_Ti_5_O_12_ followed by polypyrrole (PPy) coating to improve the electronic and ionic conductivity and thus the electrochemical performance.^11,12^

Among the different intercalation type materials, titanium–niobium oxide (TiNb_2_O_7_(3 × 3)∞, Ti_2_Nb_10_O_29_)(3 × 4)∞, etc.) shows good theoretical capacity due to the presence of multiple redox couples (Nb^5+^/Nb^4+^, Nb^4+^/Nb^3+^, Ti^4+^/Ti^3+^), and their redox potential lies within 1.0 to 2.0 V vs Li/Li^+^, which reduces dendrite growth and SEI formation.^13−16^ The two-electron reduction of niobium plays a major role in the story, despite the limitations of its higher mass, and accelerates niobium-based research for battery applications. Beyond TiNb_2_O_7_ (TNO12), which has a theoretical capacity of 388 mAh g^–1^, Ti_2_Nb_10_O_29_ (TNO15) has a higher theoretical capacity (396 mAh g^–1^) with a similar crystal structure. The Wadley-Roth crystal structure of these materials enables a shorter diffusion distance with high rate performance and lithium hopping between the corner and edge-sharing MO_6_ (M = Nb, Ti) octahedra by providing a two-dimensional interstitial spacing.^8,17,18^ Like other metal oxides, the TNO structure’s inherent low electronic conductivity (<10^–9^ S cm^–1^) limited its potential application in lithium-ion battery technology.^19,20^ There has been enormous research interest in improving the electrochemical performance, particularly the rate capability of TNO, through the coating of electrochemically active material, heteroatom doping into the TNO structure, micro- and nanostructural modification to achieve high surface area electrode materials, etc.^21−24^ The incorporation of buffer materials such as carbon coating is an important approach used to reduce volume expansion and fracture during continuous charge/discharge and improve the intrinsic electric conductivity and overall performance of bulk and nanostructured TNO anode materials. Lui et al. successfully synthesized a Ti_2_Nb_10_O_29_/C composite by the solid-state method, utilizing glucose as the carbon source, where the Ti_2_Nb_10_O_29_/C composite exhibited a better characteristic than the bare Ti_2_Nb_10_O_29_ electrode.^25^ Here, carbon has the advantage of undergoing less structural change than other materials during electrochemical performance. On the other hand, it is proven that the metal deposition, especially Au, Ag, Cu, and Ni, on the surface of the electrode material can modify the conductivity and reduce the volume expansion of the electrode material during continuous charge/discharge.^26−32^ Kim and co-workers reported that the Cu deposition on the TiO_2_ nanowire electrode material can contribute to the performance of the TiO_2_ anode material.^27^ Compared to other noble metals, Cu has excellent electric conductivity, is abundant in nature, and is low cost. Moreover, Cu is normally adopted as the current collector for lithium-ion batteries due to its stability during continuous charge/discharge cycling. Besides the modification strategies through the coating, another important approach to enhancing the electrochemical property by altering the intrinsic electronic conductivity of the material is to create oxygen vacancies in the lattice. Oxygen vacancies can be easily introduced into the Ti_2_Nb_10_O_29_ structure by calcinating the precursor in a vacuum and inert atmosphere.^33−35^ Deng et al. reported that the conductivity of Ti_2_Nb_10_O_29–*x*_ is higher than that of pristine Ti_2_Nb_10_O_29_, and the oxygen vacancies and the larger unit cell volume render the lithium diffusion path more efficient and facilitate Li-ion transportation.^36^

In this study, we successfully synthesized TNO15 microspheres through the solvothermal method and subsequent calcination in air and Ar at 800 °C for 3 h to study the role of the oxygen vacancy. In addition, glucose-derived carbon is used for metal reduction at high temperatures and nanolayer carbon coating. The obtained microspheres with an average diameter of 780 nm show a hierarchical architecture consisting of distorted primary TNO15 nanoparticles. Here we systematically investigated the change in the electrochemical performance of the prepared TNO15 microsphere by coating it with carbon and copper and the subsequent thermal reduction in the argon atmosphere. In the two-step method, the initial calcination of TNO15 precursors at 800 °C in an air and argon atmosphere resulted in the formation of monoclinic TNO15 microspheres. Further calcination at 700 °C helps with glucose reduction and the formation of uniform carbon and copper coatings on the surface of the sample. This resulted in a remarkable increase in conductivity. Benefiting from the synergistic effect of extrinsic carbon and copper coatings and intrinsic oxygen deficiency in the structure, the as-fabricated TNO15-800-C-Cu-700 electrode exhibits superior rate capacity and cycle life compared to the bare and carbon-coated samples.

## Experimental Section

### Materials

NbCl_5_ (99.0%, Sigma-Aldrich), titanium(IV) isopropoxide (>97.0%, Sigma-Aldrich), ethanol absolute (≥99.5%, VWR chemicals), acetic acid (≥99.7%, VWR Chemicals), copper(II) chloride dihydrate CuCl_2_·2H_2_O (>99.0%, Sigma-Aldrich), glucose (>99.5%, Sigma-Aldrich), LiMn_2_O_4_ (spinel (LMO) powder, battery grade, Sigma-Aldrich), 1.0 mol L^–1^ lithium hexafluorophosphate (LiPF_6_) in ethylene carbonate and dimethyl carbonate (EC/DMC ratio 1:1 with the same volume, battery grade, Sigma-Aldrich), Super P (99%, Alfa-Aesar), poly(vinylidene fluoride) (PVDF, Sigma-Aldrich), and *N*-methylpyrrolidone (NMP, anhydrous, 99.5%, Sigma-Aldrich).

### Synthesis of Ti_2_Nb_10_O_29_

Titanium niobium oxide powder was synthesized through the solvothermal method. 6.75 g of NbCl_5_ and 1.42 g of titanium(IV) isopropoxide were added to 60 mL of ethanol, and the mixing was continued with the addition of acetic acid. Once a transparent pale-yellow solution was obtained, the solution mixture was transferred to a 100 mL solvothermal setup and heated at 180 °C for 24 h. The pale-blue powder was collected and washed using water and ethanol and dried at 60 °C overnight. This sample is named as TNO15-ST. 1 g of TNO15-ST powder was further calcinated in air and argon at 800 °C for 3 h and named TNO15-800-Air and TNO15-800-Ar, respectively. Prior to the calcination in argon, the furnace was pumped down with a vacuum pump and filled with high-purity argon. Further, 1 g of the prepared TNO15-800-Air samples was mixed with 54 mg of glucose and 16 mg of CuCl_2_·2H_2_O using a pestle and mortar by adding a few drops of the ethanol/water mixture and dried at 90 °C for 30 min prior to the calcination at 700 °C in the argon flow atmosphere for 3 h to obtain TNO15-800-C-Cu-700 samples. TNO15-800-C-700 was prepared without the addition of CuCl_2_·2H_2_O. For comparison, the TNO15-C-Cu-800 sample was prepared by mixing the same quantity of glucose and CuCl_2_·2H_2_O into 1.2 g of TNO15-ST powder and calcinating at 800 °C in an argon atmosphere for 3 h.

### Material Characterization

The powder X-ray diffraction pattern of the prepared samples was recorded on a Rigaku MiniFlex 600 diffractometer equipped with Co Kα (*k* = 1.7903 Å) radiation in a range from 5 to 90° at a scan rate of 5°/min. The carbon content in the prepared samples was estimated using thermogravimetric analysis (TGA) (TA Q500, TA Instruments, USA) carried out under an air atmosphere (100 mL/min) from 25 to 800 °C with a heat rate of 10 °C/min. The morphology and nanostructure of the prepared samples were confirmed by scanning electron microscopy using Nova Nano SEM 450 and further confirmed by high resolution (scanning) transmission electron microscope FEI Titan Themis 60–300 cube, FEI, USA. X-ray photoelectron spectroscopy studies were conducted using the NEXSA-G2, monochromated high-performance XPS spectrometer, Thermo Scientific, UK, to examine the chemical composition and binding energy of the prepared samples. The nitrogen adsorption/desorption isotherms were obtained using a (BELSORP -mini II volumetric sorption analyzer) surface area and porosity analyzer.

### Electrochemical Characterization

#### N

The electrochemical testing was conducted by using a CR2023 coin cell. Briefly, the working electrode was prepared by mixing the active TNO15 and modified materials, conductive carbon black (Super P), and PVDF in a mass ratio of 8:1:1 dissolved in -methyl pyrrolidone to form a slurry. Subsequently, the prepared slurry was evenly coated on Cu foil by using the doctor blade method and dried in a vacuum at 80 °C for 12 h. The dried electrode sheet was cold pressed using a rolling machine and further cut into a circular shape with a diameter of 14 mm. The coin cells were assembled in an Ar filled glovebox with Li metal as the counter and reference electrode, 1.0 M LiPF_6_ in EC/DMC as an electrolyte, and Whatman GF/A glass microfiber filters as a separator. For the full-cell assembly, LiMn_2_O_4_ (LMO) was used as the cathode material and coated on the Al foil, and TNO15-800-C-Cu-700 was used as an anode. The half-cell and full-cell were tested in a voltage range of 1.1 to 2.5 V and 1.5 to 3.2 V, respectively, using a Bio-Logic BCS-810 battery cycler at room temperature.

## Results and Discussion

**Figure 1:**
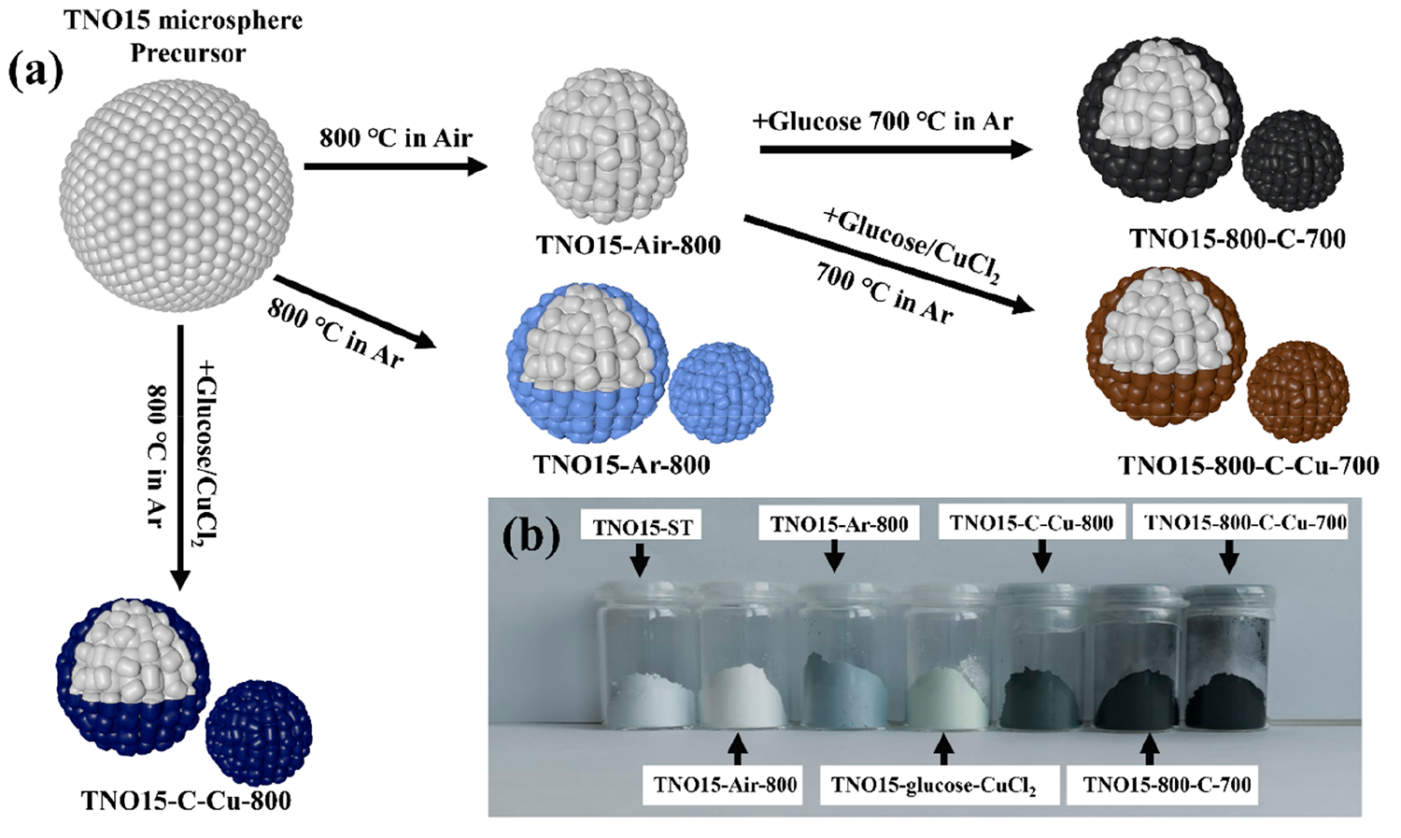
(a) Illustration of the synthesis of TNO15 and its modified samples and (b) photographs of prepared TNO15 and modified samples.

A schematic illustration of the synthesis process of TNO15 and the modified samples is shown in Figure 1(a). First, titanium(IV) isopropoxide and NbCl_5_ in an ethanol/acetic acid mixture at 180 °C for 24 h in solvothermal conditions results in the formation of a TiO_2_/Nb_2_O_5_ mixture. Further, the initial calcination temperature was chosen according to the previous literature reports, where calcination at 800 °C eliminates the formation of anatase-TiO_2_ and Nb_2_O_5_ in the TNO15 sample.^37^ The as-prepared TNO15-ST powder is pale blue in color. After the calcination in air and argon atmospheres, the sample changed its color to pure white and light blue, respectively. The photograph of the prepared samples is shown in Figure 1(b). The clearly observable color difference in the samples calcinated in air and argon atmospheres is associated with the reduction of Nb^5+^ to Nb^4+^ in addition to the presence of a Ti^4+^/Ti^3+^ mixed state. It has already been reported that the calcination of niobium-based oxide in a reducing atmosphere induces partial reduction of Nb^5+^ to the Nb^4+^ state and results in a color change to dark blue.^33,35^ Compared to the TNO15-Ar-800 sample, the Prussian blue color of TNO15-C-Cu-800 is associated with a higher reduction of niobium in the presence of a carbonaceous material at a high temperature of 800 °C. The photographs of the prepared TNO15-Air-800, TNO15-Ar-800, and TNO15-C-Cu-800 are shown in Figure S1 for comparison. Here, glucose acts as a reducing agent. Cu^2+^ has already been reduced to Cu^+^ at 90 °C seen from the light green color in TNO15-glucose-CuCl_2_-90. Further calcination of these samples at 700 °C offers a uniform coating of carbon and copper on the surface of TNO15 in addition to the formation of lower valence cations. The carbon-coated TNO15 sample is black, and the carbon/coppercoated sample is dark brown due to the presence of Cu metal. More studies on surface reduction and oxygen vacancies in the prepared samples are included in the TGA and XPS sections.

**Figure 2:**
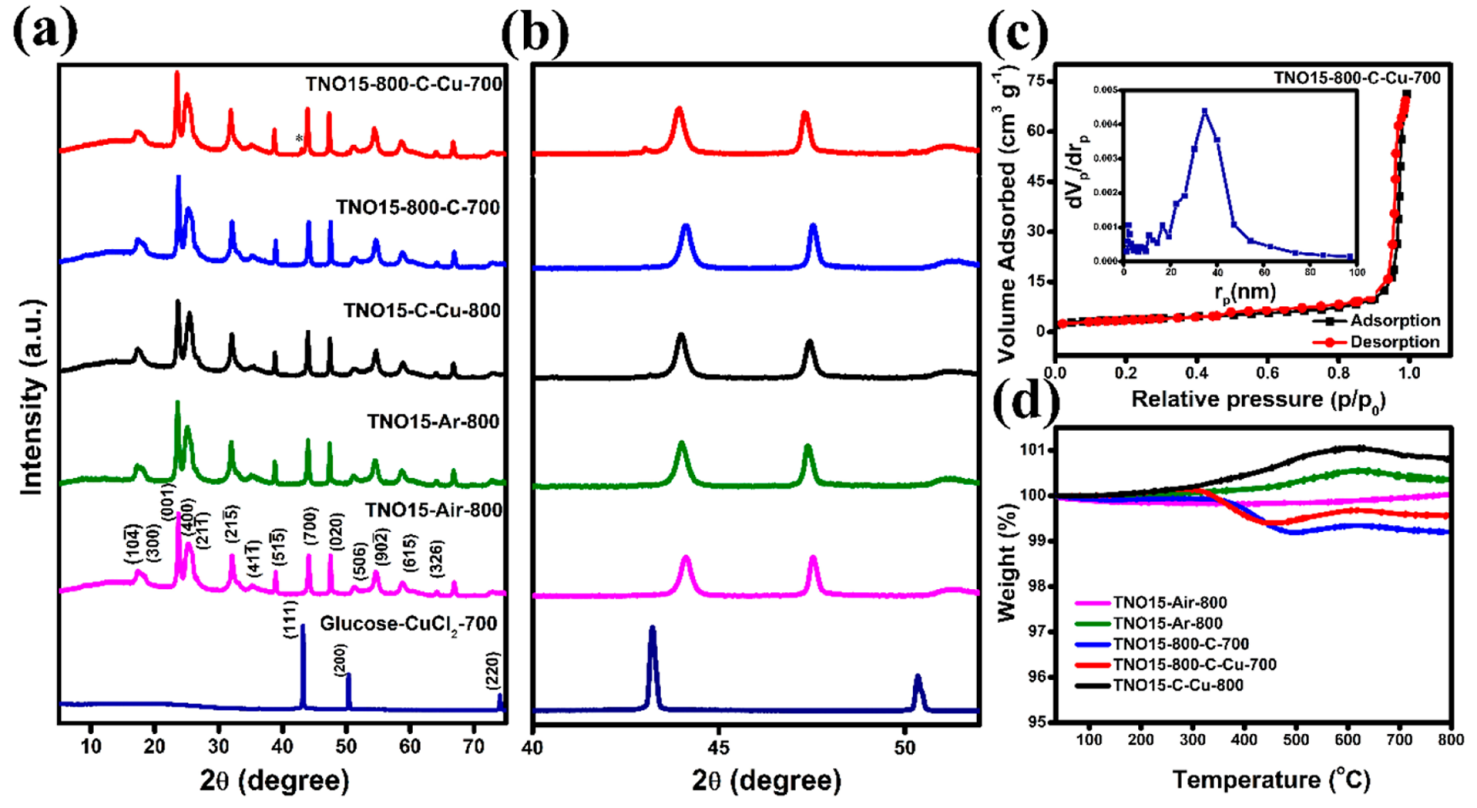
(a) XRD analysis of the TNO15, glucose-CuCl_2_-700, and modified samples, (b) XRD pattern in the 2θ range of 40–55°, (c) BET and BJH (inset) results of the TNO15-800-C-Cu-700 sample, and (d) TGA analysis of prepared samples.

The XRD pattern for the TNO15 precursor obtained after the solvothermal synthesis is shown in Supporting Information, Figure S2(a). The patterns correspond to JCPDS No. 21-1272 and 43-1042 for TiO_2_ and Nb_2_O_5_ respectively. This confirms that the solvothermal synthesis results in the nucleation and growth of TiO_2_/Nb_2_O_5_ mixed structures, and during the calcination at 800 °C, the formatted TiO_2_/Nb_2_O_5_ mixture turns into Ti_2_Nb_10_O_29_. After calcination at 800 °C, no detectable Nb_2_O_5_ or TiO_2_ phases can be found in the XRD pattern of the prepared TNO15 and modified samples, as shown in Figure 2(a). The obtained diffraction pattern matches with the JCPDS card No. 72–0159, implying a complete monoclinic Ti_2_Nb_10_O_29_ crystallographic system. The diffraction peaks of carbon cannot be detected in the XRD pattern, which could be attributed to the low content and amorphous nature of the coated carbon in the sample. The XRD pattern of the glucose/CuCl_2_·2H_2_O mixture calcinated at 700 °C (sample code glucose-CuCl_2_-700) shows the formation of Cu metal through the reduction of CuCl_2_ by glucose, and the peaks observed at 43.1, 50.5, and 74.3° correspond to the (111), (200), and (220) planes of Cu (JCPDS No. 04–0836) respectively. The XRD pattern of TNO15-800-C-Cu-700 and TNO15-C-Cu-800 samples shows a weak diffraction peak at 43°, shown in Figure 2(b), confirming the presence of Cu metal in the samples. There is no shift in the diffraction peaks for the carbon and carbon/copper-coated samples, purely indicating there is no unit cell volume expansion occurring like the doping of metal. The XRD analysis of the glucose-CuCl_2_ mixture after drying at 90 °C (sample code glucose-CuCl_2_-90) is shown in Figure S2(b). From the XRD pattern, at 90 °C, glucose reduces CuCl_2_ to CuCl, and the pattern matches JCPDS No. 06–0344 and we observed a color change in the sample to light green.

To measure the specific surface area and the pore size distribution of the prepared samples, a N_2_ adsorption/desorption experiment was conducted. The corresponding BET and BJH results are illustrated in Figures 2(c) and S3. The as-prepared TNO15 and modified samples exhibit a type IV isotherm with an average pore radius <50 nm. This mesopore mainly resulted from the voids between the interconnected TNO15 primary particles, as shown in the SEM results. From the results, the TNO15-800-C-Cu-700 sample exhibits a specific surface area of 12.9 m^2^ g^–1^, which is slightly higher than those of the TNO15-Ar-800 (11.8 m^2^ g^–1^), TNO15-800-C-700 (10.8 m^2^ g^–1^), and TNO15-C-Cu-800 (11.7 m^2^ g^–1^) samples. The TNO15 calcinated in air at 800 °C shows a higher specific surface area of 63 m^2^ g^–1^. The reduction in the specific surface area of the carbon/copper-coated samples is due to their layer deposition on the pore channels, and similar results were reported previously.^38^ The average pore volume of the TNO15-Ar-800 sample (48.6 nm) is larger than that of the TNO15-Air-800 (43.2 nm) sample. Thus, the reduction in the specific surface area of TNO15-Ar-800 compared to the sample calcined in air is mainly due to the collapse of smaller diameter pores. Table S1 in the Supporting Information compares the BET surface area, pore volume, and pore size of the prepared samples.

The composition content of the carbon and the presence of oxygen vacancies in the prepared samples were ascertained by the thermogravimetric analysis under air atmosphere, and the results are compared in Figure 2(d). A weight gain is observed at 200 °C for all the samples other than TNO15-Air-800 due to the removal/vanishing of oxygen vacancy, and is continued for TNO15-Ar-800 and TNO15-C-Cu-800 until 600 °C. The weight gain is higher at 250 °C for TNO15-800-C-Cu-700 than for TNO15-800-C-700 due to the formation of CuO. The weight loss observed at 300 °C in these samples confirms the decomposition of carbon, and the calculated carbon content is 0.8 and 0.6% in TNO15-800-C-700 and TNO15-800-C-Cu-700, respectively. The TGA curve of the TNO15-C-Cu-800 shows no carbon reduction due to the absence or negligible amount of carbon in this compared to the other two carbon-coated samples. This occurs because most of the carbon is used to reduce niobium at high temperature of 800 °C. A similar reduction was observed by us in our previous study, where the reduction of niobium oxide is high at higher temperatures in the presence of carbon.^39^ Hence, with the support of this study and the TGA analysis here, we confirmed that the reduction of metal is high at high temperatures. The dark blue color of the sample calcinated at 800 °C (Figure S1) compared to that of the other samples confirms the above statement. In addition, the higher weight gain of this sample compared to others is due to the higher oxygen vacancy associated with the presence of lower valence cations. The electrical conductivity of the prepared samples was tested using four-probe measurements at room temperature, and the results are provided in Table S2 in the Supporting Information. TNO15-800-C-Cu-700 and TNO15-800-C-700 have high conductivity, on the order of ∼10^–3^ S cm^–1^, which is much higher than the TNO15-Air-800 and TNO15-Ar-800 samples. The conductivity of TNO15-C-Cu-800 is lower due to the lesser amount of carbon in the sample.

**Figure 3:**
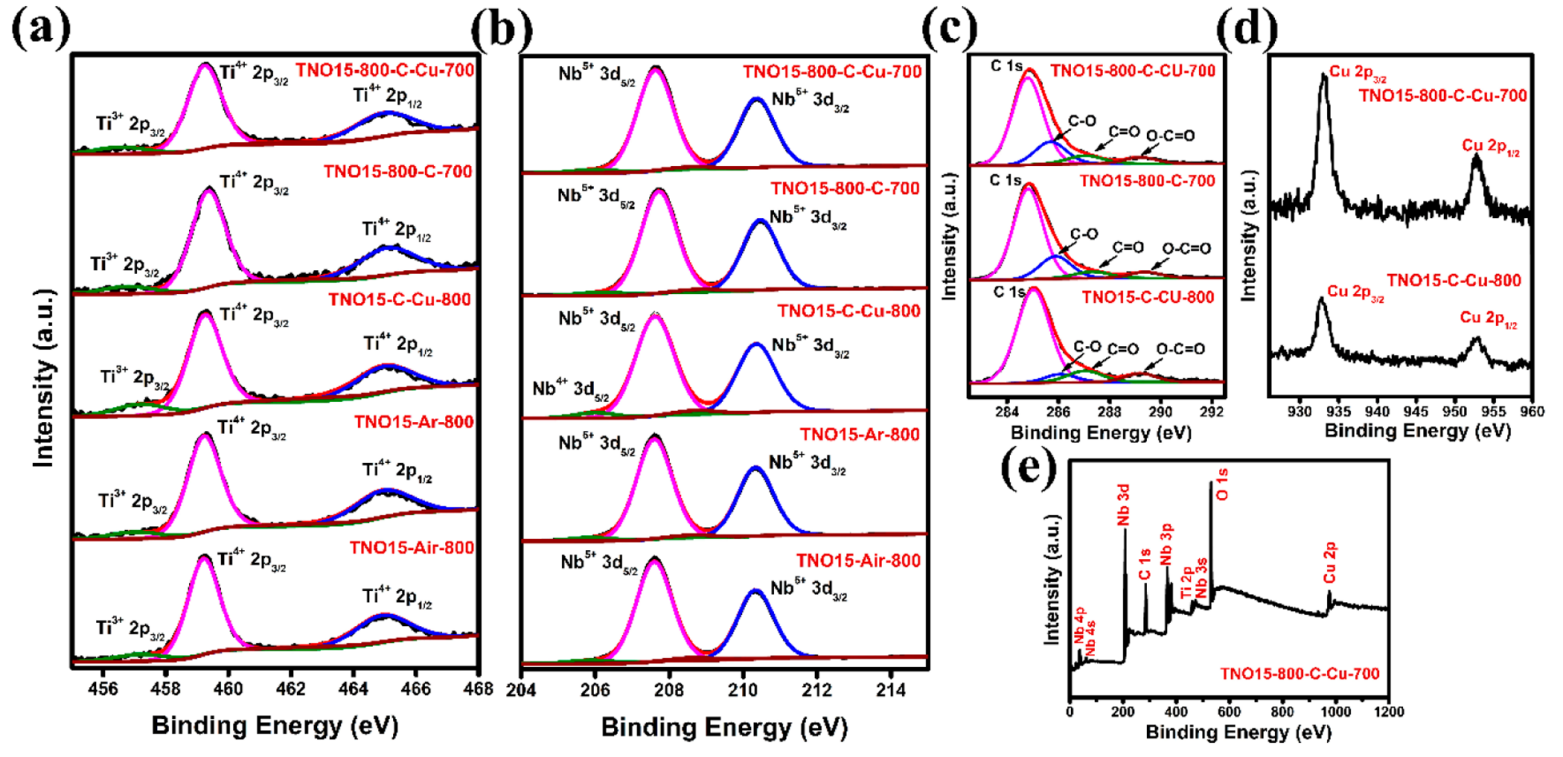
XPS results of the TNO15 and modified samples. XPS spectra of the (a) Ti 2p state, (b) Nb 3d state, (c) C 1s state in the samples as well as (d) the Cu 2p state and (e) survey spectrum for the TNO15-800-C-Cu-700 sample.

Further, the stoichiometric composition and chemical valence state of the Ti, Nb, O, C, and Cu in the prepared TNO15 and modified powder samples were analyzed by X-ray photoelectron spectroscopy, and the obtained results are shown in Figure 3. The resolved Ti 2p spectra for all of the prepared samples are shown in Figure 3(a). The binding energy doublet peaks located at 459.3 and 464.7 eV with a binding energy separation of 5.5 eV confirm the presence of Ti 2p_3/2_ and Ti 2p_1/2_ states, respectively.^40^ However, with the major Ti^4+^ peaks, minor shoulder peaks observed at 457.5 eV were attributed to the presence of the Ti^3+^ oxidation states in the samples. From the peak area analysis, the Ti^3+^ to Ti^4+^ ratio is higher in TNO15-C-Cu-800 than in the sample calcinated in argon and air at 800 °C and in carbon and carbon/coppercoated samples at 700 °C. From this, it is clear that, in addition to the reduction using carbonaceous material, the choice of calcination temperature is also a critical factor for the creation of lower valence cations and oxygen vacancies in the Ti_2_Nb_10_O_29_. The high-resolution XPS spectrum for the Nb^5+^ 3d state in the prepared TNO15 samples is shown in Figure 3(b). The observed binding energy doublets at 207.5 and 210.2 eV correspond to 3d_3/2_ and 3d_5/2_ states, respectively. The deconvoluted Nb 3d spectrum of TNO15-C-Cu-800 shown in the Supporting Information, Figure S4(a), points out the presence of Nb^4+^ 3d_3/2_ and 3d_5/2_ doublets with binding energy values of 208.8 and 205.9 eV, respectively. The high-intensity Nb^4+^ doublets observed in TNO15-C-Cu-800 compared to the TNO15-800-C-Cu-700 sample imply a higher reduction percentage of the Nb^5+^ state to Nb^4+^. The peak area ratios of Ti^3+^/Ti^4+^ and Nb^4+^/Nb^5+^ are given in the Supporting Information Table S3. The Ti^4+^ and Nb^5+^ spectra of TNO15-800-C-700 slightly shift to higher binding energy values by 0.15 and 0.12 eV, respectively, due to the presence of carbon in the sample.^41,42^ However, there is no shift in the binding energy values of the other two carbon-coated samples, probably due to the lesser amount of carbon compared to TNO15-800-C-700. The O 1s spectrum for the prepared samples is shown in Figure S4(b) and is divided into two. The peak corresponding to the lattice’s oxygen/metal–oxygen bond is located at 530.2 eV, and the peaks at 532.28 eV represent the physi- and chemisorbed water at or near the surface.^43,44^ Unlike the other reports, we were unable to find the oxygen defect or vacancy peaks in the O 1s spectrum.^45,46^ The deconvoluted C 1s spectra for the TNO15-800-C-700, TNO15-C-Cu-800, and TNO15-800-C-Cu-700 samples are shown in Figure 3(c). The dominant peak located at 284.6 eV corresponds to C–C/C=C, and the minor peaks at 285.6, 287.3, and 289.2 eV represent C–O, C=O, and O–C=O, respectively.^47^Figure 3(d) shows the high-resolution XPS data recorded for the Cu 2p state. Here, the Cu 2p energy levels of TNO15-800-C-Cu-700 and TNO15-C-Cu-800 were composed of characteristic doublets corresponding to Cu 2p_3/2_ and Cu 2p_1/2_ at 933.08 and 952.8 eV, respectively. The absence of satellite peaks in the energy spectra of both samples implies a filled d orbit and confirms the presence of Cu metal in the sample. Figure 3(e) shows the XPS survey spectrum for the TNO15-800-C-Cu-700 sample with a binding energy value of 0–1200 eV. The characteristic binding energy peaks corresponding to oxidation states in the full spectrum confirm the existence of Ti, Nb, C, O, and Cu on the surface of the sample.

**Figure 4:**
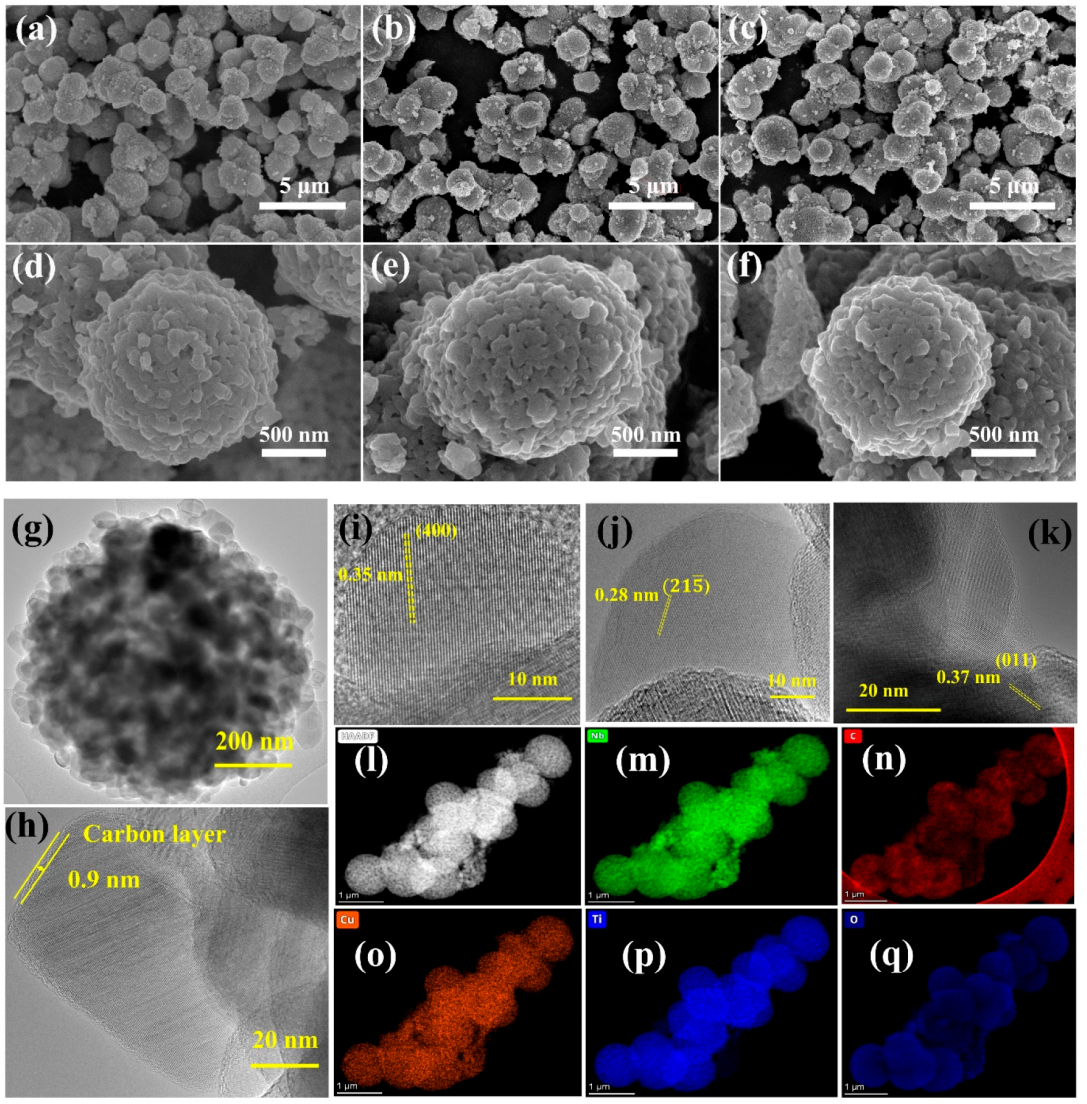
Morphology characterization of the prepared TNO15 and modified samples. SEM images of (a,d) TNO15-Air-800, (b,e) TNO15-800-C-700, and (c,f) TNO15-800-C-Cu-700 at different magnifications, (g–k) TEM images of TNO15-800-C-Cu-700, and (l–q) HAADF elemental mapping for the TNO15-800-C-Cu-700 sample.

The micro/nanostructures of the prepared TNO15 and modified samples were studied using SEM and TEM analysis. Figure 4(a–f) shows the high- and low-magnification SEM images of TNO15-Air-800 (Figure 4(a,d)), TNO15-800-C-700 (Figure 4(b,e)), and TNO15-800-C-Cu-700 (Figure 4(c,f)) samples. The SEM images of TNO15-Ar-800 and TNO15-C-Cu-800 are provided in the Supporting Information, Figure S5(a–d). Here, the microsphere structure is formed by the self-assembly of TNO15 nanoparticles via solvothermal synthesis, followed by calcination. This secondary microsphere displayed an average diameter of 780 nm. From these results, it is obvious that the formation of a secondary microsphere through the interconnected TNO15 primary nanocrystals enables a porous network and contributes to the Li-ion transportation during the electrochemical reaction. To confirm the presence of Cu on the surface of the prepared TNO15-800-C-Cu-700, SEM-EDX spectroscopy analysis is conducted, and the result is shown in Figure S6. The peaks presented in the spectrum confirm the existence of Ti, Nb, C, O, and Cu on the surface of the sample. The SEM images of TNO15 precursors obtained from the solvothermal synthesis are shown in Supporting Information, Figure S7.

Further, the nanothick carbon and copper deposition on the TNO15 microsphere is confirmed with TEM, HRTEM, and HAADF analysis and shown in Figure 4(g–q). Figure 4(g–k) shows the TEM and HRTEM images of the TNO15-800-C-Cu-700 sample. As shown in Figure 4(h), the average thickness of the carbon layer on the TNO15 nanoparticles is 0.9 nm. The lattice spacing in the HRTEM images shown in Figure 4(i–k) is determined to be 0.35, 0.28, and 0.37 nm, which is in good agreement with the (400), (215), and (011) crystallographic planes of monoclinic TNO15, respectively. The TEM and HRTEM images of TNO15-800-C-700 and TNO15-C-Cu-800 samples are shown in Figures S8(a,b) and S8(c,d), respectively. This confirms the distorted spherical morphology formed with TNO15 primary nanocrystals. The nanolayer carbon coating on the surface of the TNO15-800-C-700 sample is confirmed from the HRTEM images shown as Figure S8(b). In TNO15-C-Cu-800, the reduction of carbon is higher, and we are unable to observe the coated carbon on the surface of the TNO15 primary nanostructures, as shown in Figure S8(d). The presence of Cu on the surface of TNO15 samples is confirmed using STEM-HAADF elemental mapping. The STEM-HAADF mapping result for the TNO15-800-C-Cu-700 sample is shown in Figure 4(l–q). In this, we confirmed the even distribution of Nb (Figure 4(m)), carbon (Figure 4(n)), and copper (Figure 4(o)) in addition to Ti (Figure 4(p)) and O (Figure 4(q)) on the surface of the prepared TNO15. For additional confirmation, the selected area elemental mapping and elemental spectrum for TNO15-800-C-Cu-700 are provided in Figure S9(a,b), respectively. This will further support the presence of C, O, Ti, Cu, and Nb on the surface of the sample. Figure S9(c–f) shows different HAADF images, and the corresponding Cu elemental mapping authenticates the presence of Cu on the surface of the sample.

**Figure 5:**
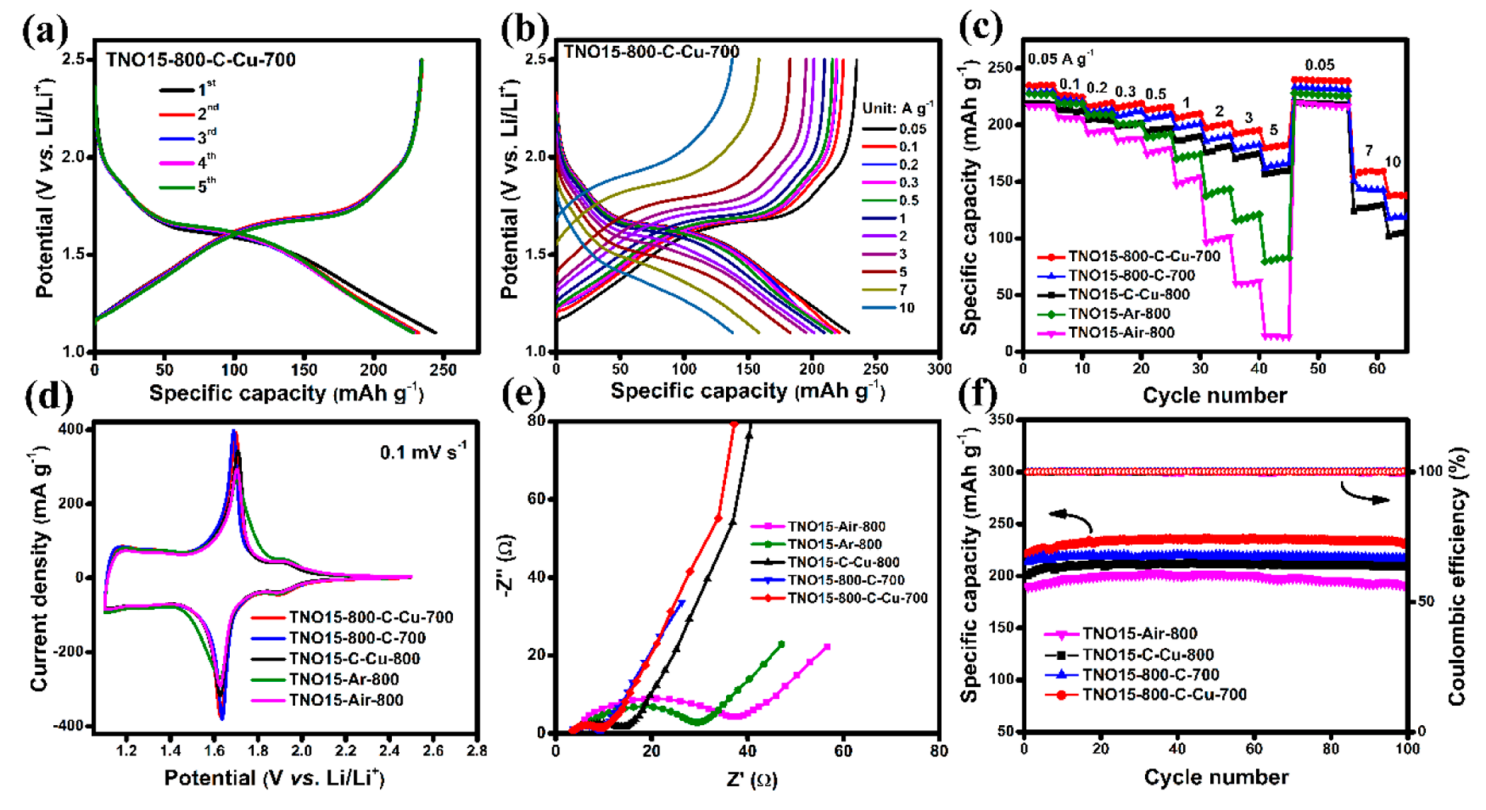
Electrochemical performance of TNO15 and modified samples. (a) Initial five charge/discharge curves of TNO15-800-C-Cu-700 at 0.05 A g^–1^ current density, (b) charge/discharge curve of TNO15-800-C-Cu-700 at different current densities, (c) rate performance of TNO15 and modified samples at different current densities, (d) comparative CV performance of the prepared electrodes at a 0.1 mV s^–1^ sweep rate, (e) electrochemical impedance performance of the prepared half-cells, (f) comparative stability performance and Coulombic efficiency for the prepared electrodes at 0.2 A g^–1^ current density.

The electrochemical performance of the prepared TNO15 and modified samples was characterized by half-cell assembly using the lithium metal reference/counter electrode within a potential range of 1.1 to 2.5 V. The initial five curve charge/discharge profile for the TNO15-800-C-Cu-700 electrode at 0.05 A g^–1^ current density is shown in Figure 5(a). As reported previously, this profile can be classified into three regions. The first region, where the voltage drops steeply from 2.5 to 1.7 V vs Li/Li^+^, is considered the initial Li-intercalation region.^37,48^ This steep slope is generally associated with the formation of a single solid solution phase. Here in the region, the slope observed at ca. 1.9 V vs Li/Li^+^ is generally referred to as the reduction potential of Ti^3+^/Ti^4+^. However, Guo et al. found that there is a simultaneous reduction of Ti^4+^ to Ti^3+^ from 3.0 to 1.0 V vs Li/Li^+^, while Nb^5+^ ions are continuously reduced to Nb^4+^ from 3.0 to 1.6 V vs Li/Li^+^ and further reduced to Nb^3+^ from 1.6 to 1.0 V.^49^ Thus, it is concluded that this charge/discharge plateau should signify the average redox potential of Ti and Nb.^17,19,49^ The second region from 1.7 to 1.6 V vs Li/Li^+^ is the plateau corresponding to the coexistence of the two-phase reaction process. Wu et al. further confirmed the existence of a two-phase reaction region using in situ XRD analysis.^48^ This region is normally characterized by a slower lithium diffusion rate and a high degree of lithiation.^50^ Finally, there is a long sloping region (region 3) from 1.6 to 1.1 V vs Li/Li^+^ revealing different lithium insertion behaviors, including capacitive charge storage mechanisms. Here, the TNO15 and modified electrodes provide a reasonably high working window, which assures safety during the lithium intercalation/deintercalation processes, and the cutoff voltage of 1.1 V was chosen to avert the formation of any kind of electrolyte interphase. As illustrated in Figure 5(a), the initial charge capacity for TNO15-800-C-Cu-700 was obtained as 234.1 mAh g^–1^ with 95.88% Coulombic efficiency. After the five charge/discharge cycles, the electrode maintained 100% efficiency, indicating that the electrode material possesses excellent reversible kinetics. In contrast, for the other working electrodes, TNO15-Air-800, TNO15-Ar-800, TNO15-C-Cu-800, and TNO15-800-C-700, the initial charge capacity (initial Coulombic efficiency) is 221.9 mAh g^–1^ (90.86%), 229.16 mAh g^–1^ (93.10%), 219.9 mAh g^–1^(95.1%), and 229.92 mAh g^–1^ (94.15%), respectively, at 0.05 A g^–1^ current density. After five cycles, except for TNO15-Air-800, other electrodes maintain 100% Coulombic efficiency owing to their surface reduction and modification. The first five charge/discharge performances of the electrode materials are compared and are presented in Figure S10. From these performances, we observed an irreversible loss in capacity during the initial charge/discharge, which is mainly due to the insertion of the Li^+^ residue into the distorted lattice structure of TNO15.^19^ It is noteworthy that the performance of the TNO15-800-C-Cu-700 electrode has the highest specific capacity and Coulombic efficiency compared to the other electrodes. The uncoated TNO15 microspheres, composed of a large number of primary nanocrystallites, create a high degree of discontinuities between the particle boundaries, which slow down the Li-ion charge/discharge kinetics and reduce the intrinsic ionic conductivity of the material. By layering the carbon on the primary TNO15 nanoparticles, they act as a bridge between the nearby nanocrystallites and enable better electron/ion transportation through the structure. Simultaneously, the formed oxygen vacancies in the TNO15 structure create an addition reaction site that helps for better Li^+^ diffusion. Furthermore, the presence of metallic Cu on the surface of the TNO15 nanocrystallites enables a high extrinsic electronic conductivity and better ion transportation. From this, it is evident that in addition to the carbon/copper coating on the surface of TNO15, the oxygen vacancy plays a significant role in the performance of the electrode material. Previous studies done by Goodenough et al. provided that the addition of carbon on the surface of the TNO12 electrode not only improves the extrinsic electronic conductivity of the electrode material but also contributes to the electrochemical performance and stabilizes the Nb(IV) valence state.^13^ Rate capability is another important parameter for the operation of the electrode at high current densities. The charge/discharge performance for TNO15-800-C-Cu-700 was further tested at higher current densities from 0.1 to 10 A g^–1^ (Figure 5(b)). Prior to the charge/discharge performance, all of the prepared cells were charged/discharged at 0.05 A g^–1^ current density for five cycles. At a 0.1 A g^–1^ current density, the reversible capacity of the TNO15-800-C-Cu-700 electrode is reduced to 225.8 mAh g^–1^, and when the current density is increased to 0.5 A g^–1^, 94.6% of the initial capacity is maintained. Further, the current density is increased to 1, 2, 5, and 10 A g^–1^, the reversible capacity is 206.3, 197.4, 179.7, and 138 mAh g^–1^, respectively, and maintained 100% Coulombic efficiency at 10 A g^–1^. It is significantly higher than that of TNO15-C-Cu-800 and TNO15-800-C-700 with 105.8 and 117.8 mAh g^–1^ capacity and 96.3 and 98.74% Coulombic efficiency at 10 A g^–1^ current density, respectively.

To explore the structural evolution of the TNO15-800-C-Cu-700 electrode, in situ XRD analysis was recorded and is shown in Figure S11. The analysis was conducted by fabricating a CR2032 coin cell with a Kapton window, and the XRD patterns were recorded at selected potentials during the charge/discharge between 2.5 and 1.1 V at 0.1 A g^–1^ current density. The electrode shows a significant shift in the 2θ corresponding to (011), (400), (215), (515), (700), (020), (902), and (615) planes during lithiation/delithiation with good reversibility. This indicates a pseudocapacitive charge storage mechanism with a change in the lattice parameter. In addition to this, the appearance of a new minor peak at 51.5° in the two-phase coexistence potential region points out that there is a formation of a new phase or a crystallographic phase transition during this potential scan. This in situ XRD analysis validates that the charge storage mechanism in TNO15-800-C-Cu-700 is the combination of lithium intercalation with dominant pseudocapacitance, where Li^+^ is inserted into the layers and tunnels of the active material, followed by Faradaic charge transfer.

The charge/discharge rate performances of the prepared half-cells are compared in Figure 5(c). When the current density is returned from 5 to 0.05 A g^–1^, the capacity of TNO15-800-C-Cu-700 is increased back to 239.9 mAh g^–1^. For the bare sample TNO15-Air-800, when there is a hundred-fold increment in the current density (from 0.05 to 5 A g^–1^), a drastic drop in the charge capacity to 13.7 mAh g^–1^ is observed. However, the material is quite good at maintaining a Coulombic efficiency of 95.6%. Coming to the TNO15-Ar-800 electrode, at higher current densities, the drop in capacity (73 mAh g^–1^ at 5 A g^–1^ current density) is higher compared to the carbon and carbon/coppercoated samples. This points out how the improvement in electronic conductivity contributes to better performance of the electrode material during the charge/discharge. Here, the electrode TNO15-800-C-Cu-700 thankfully maintains 60% of its initial capacity even at a higher current density of 10 A g^–1^. It is reasonably higher than those of TNO15-800-C-700 (51% at 10 A g^–1^) and TNO15-C-Cu-800 (46.5% at 10 A g^–1^) electrodes. Unlike the difference in the reversible capacity, there is no change observed in the shape of the charge/discharge curves, which implies that without affecting the fundamental charge storage mechanism of TNO15, the carbon/copper coating improves the performance. From the obtained results, the electrochemical performance of TNO15-800-C-Cu-700 is better than that of the TNO15-C-Cu-800 electrode. As discussed in the previous sections, the presence of oxygen vacancies and lower valence cations is higher in the TNO15-C-Cu-800 electrode compared to the TNO15-800-C-700 and TNO15-800-C-Cu-700, thus increasing the intrinsic conductivity. However, from our studies, it is evident that, for enhanced Li-storage and Li-transportation properties, carbon and metal coating is a better method than creating oxygen vacancies.

The redox process during charge/discharge was evaluated by cyclic voltammetry (CV) studies. The comparative CV curves for the TNO15-Air-800, TNO15-Ar-800, TNO15-C-Cu-800, TNO15-800-C-700, and TNO15-800-C-Cu-700 electrodes at a 0.1 mV s^–1^ sweep rate are shown in Figure 5(d). A pair of redox peaks observed at ca. 1.68 V vs Li/Li^+^ (oxidation peak) and ca. 1.64 V vs Li/Li^+^ (reduction peak) in the CV curves correspond to the voltage plateaus shown in Figure S10, which are attributed to the Nb^5+^/Nb^4+^ redox couple.^48^ Hence, the average working potential of the cell is ca. 1.66 V vs Li/Li^+^, and such a high potential avoids electrolyte decomposition and the formation of electrolyte interfaces. Compared to the CV curve of the other electrode, the anodic and cathodic peak shift in the TNO15-800-C-Cu-700 is reduced to 0.054 V, indicating smaller polarization and better reaction kinetics.^51^ At a lower sweep rate of 0.1 mV s^–1^, the current response of the TNO15-800-C-700 electrode is slightly higher than that of the TNO15-800-C-Cu-700 electrode. With an increase in the current density, the TNO15-800-C-Cu-700 electrode maintained a higher capacity retention than the carbon-coated electrode. This shows how the copper deposition helps to improve the performance of the electrode more than the pristine one. Previously, Suzuki and co-workers studied Li transportation through metallic copper and proved that without any side reactions, the coated Cu layer eases Li transfer.^52^ In addition, by comparing the peak intensity and peak position of the bare, carbon, and carbon/copper-coated electrodes, it is obvious that the coating enhances the reversibility of the electrode material. Further, the appearance of the rectangular area below 1.55 V vs Li/Li^+^ points out the electrochemical features of capacitance together with the reduction of Nb^4+^/Nb^3+^.^44,53^

Electrochemical impedance spectroscopy (EIS) was conducted to understand the charge-transfer kinetics at various parts of the electrode system with a small perturbing sinusoidal voltage of amplitude of 10 mV. Figure 5(e) shows the Nyquist plot for the prepared electrodes within a frequency range of 0.01 Hz to 10 kHz. All the electrode systems follow a common trend of a depressed semicircle in the high-medium frequency region followed by a straight line in the low-frequency region. The initial *x*-intercept in the high-frequency region provides information about the contact resistance that arises from the electrolyte, separator, and electrode, called the ohmic impedance. The semicircle in the high- to medium-frequency region represents the complex reaction process over charge transfer, and the diameter of the semicircle provides information about the charge-transfer resistance. The linear line in the lower frequency region (called the Warburg impedance) corresponds to lithium diffusion through the electrodes. As shown in Figure 5(e), the ohmic impedance of the carbon and carbon/coppercoated samples was less than TNO15-Air-800 and TNO15-Ar-800. In the high- to medium-frequency region, the diameter of the semicircle was reduced for TNO15-800-C-700, TNO15-C-Cu-800, and TNO15-800-C-Cu-700 electrodes due to better charge-transfer kinetics, such as rapid electron and lithium transport. This indicates that the coating on the TNO15 surface improves charge transfer and boosts reaction kinetics. Moreover, the Warburg impedance corresponding to lithium diffusion was improved for the carbon and carbon/coppercoated electrodes.

Including the high capacity and rate capability, long-term stability is an important factor for lithium-ion batteries. In Figure 5(f), the charge capacities of the TNO15-Air-800, TNO15-800-C-700, TNO15-C-Cu-800, and TNO15-800-C-Cu-700 electrodes are plotted for 100 cycles at a current density of 0.2 A g^–1^. Here, the carbon and copper coating on the electrode surface reduces the structural deformation during the electrochemical charge/discharge. All of the electrode materials maintain 100% Coulombic efficiency at the end of the 100th cycle except TNO15-Air-800. Here, we observed a slight increment in the capacity at the first 20 cycles, and the capacity is maintained until 100 cycles. This initial increment in capacity is due to the activation of electrochemically active sites in the electrode material and electrolyte into smaller pores. Good cycling stability comes from the structure of TNO15 and the potential window, in addition to the improved structural stability and electronic and ionic transport of the carbon and carbon/coppercoated electrodes to the bare electrodes.

**Figure 6:**
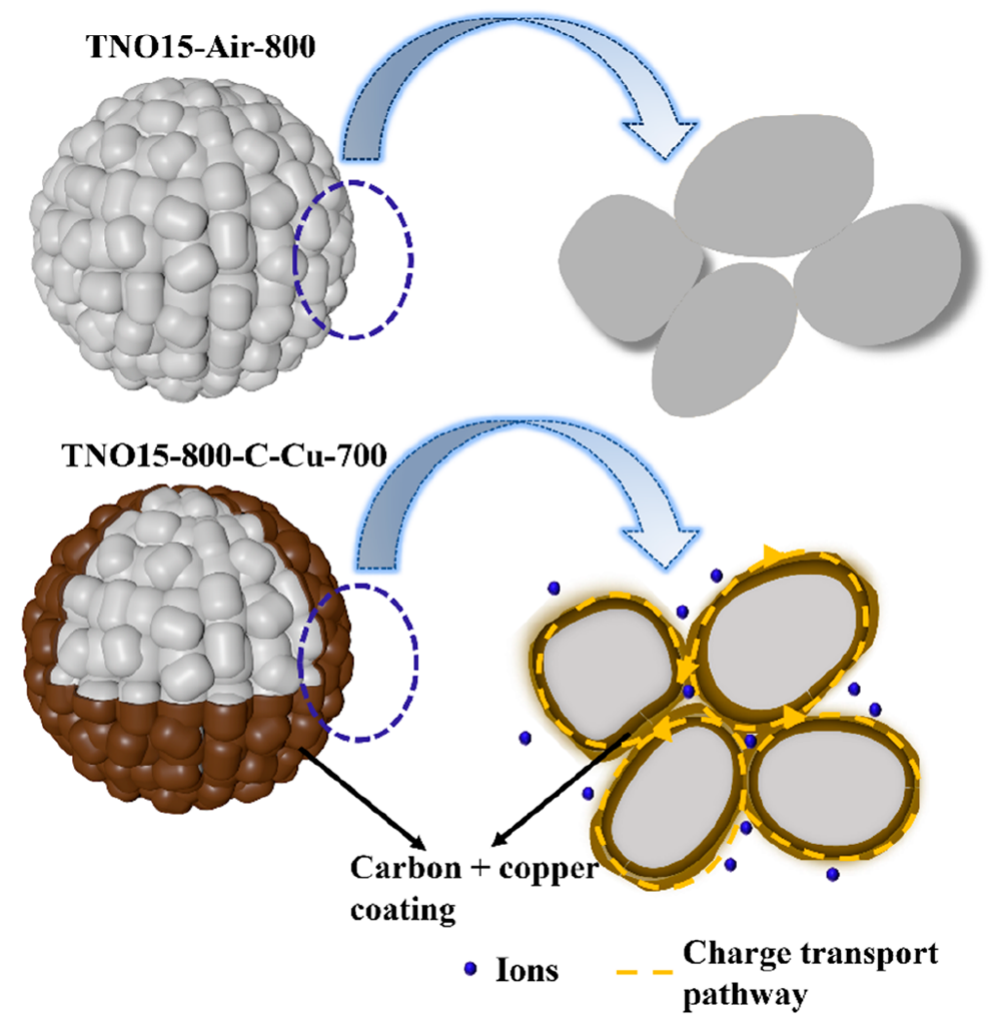
Illustration representing how the carbon/copper coating influences charge transportation in TNO15-800-C-Cu-700.

The mechanism of electrochemical charge storage and structural design are illustrated in Figure 6. As discussed previously, the prepared TNO15 exhibits a 3D intercalated porous array structure formed from the primary TNO15 nanocrystallite, and the gap between the primary nanocrystallites diminishes the electron/charge migration. Here, nanolayer carbon and copper coatings on the surface of the TNO15 nanoparticle reduced the energy barrier for Li^+^ transportation and eased the diffusion during charge/discharge. The nanolayer carbon coating on the electrode surface enables the utilization of active areas of the electrodes in addition to the improvement in the extrinsic electronic conductivity provided by the carbon/copper coating on the surface. Thus, integrating highly conductive copper metal with carbon on the surface of TNO15 facilitates a reduction in the charge-transfer resistance and provides more active areas, better rate capability, and electrochemical performance. In addition, it has already been reported that the overall electronic conductivity is limited by lower carbon content, while increasing carbon content reduces the utilization of active material and reduces performance.^54^ Thus, the optimum carbon content or carbon layer thickness is a critical factor in obtaining excellent energy storage performance for TNO15 samples.

**Figure 7:**
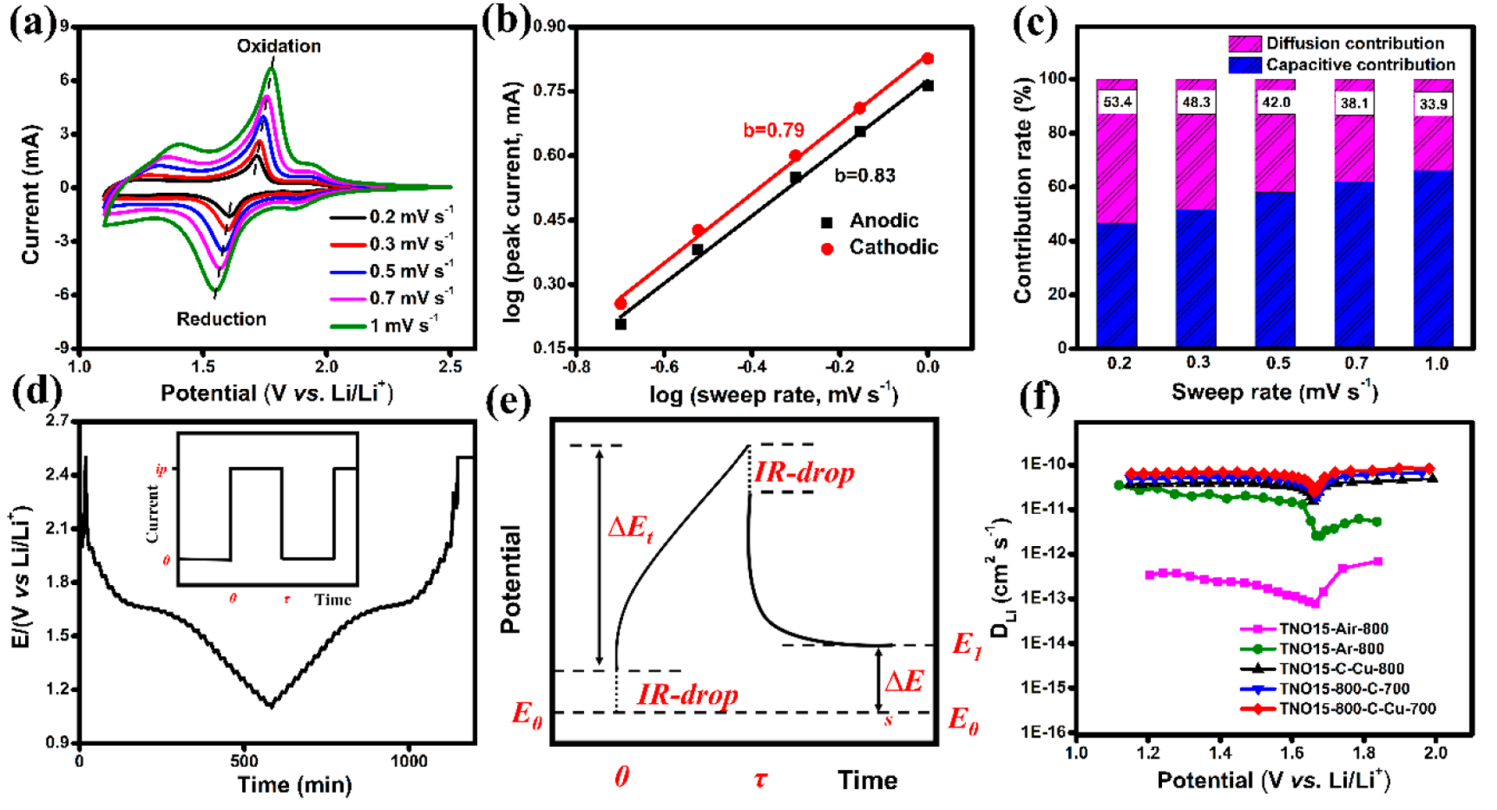
(a) CV performance of TNO15-800-C-Cu-700 at different sweep rates from 0.2 to 1 mV s^–1^, (b) fitted log (peak current) vs log (sweep rate) curve for TNO15-800-C-Cu-700, (c) capacitive and diffusion contributions to the total current at various sweep rates for the TNO15-800-C-Cu-700 half-cell, (d) GITT experimental data for TNO15-800-C-Cu-700 (inset scheme of current vs time of a single GITT measurement), (e) scheme of a single GITT measurement, and (f) diffusion coefficient vs potential graph for the prepared electrodes.

The CV performance of the TNO15-800-C-Cu-700 electrode with a sweep rate (υ) is shown in Figure 7(a). With an increase in sweep rates, the anodic and cathodic peaks become broad with an increase in the peak current, and a noticeable separation (Δ*E*_p_) is observed in the peak position. This is attributed to the polarization (related to the higher overpotentials necessary to deliver the higher current) during the charge/discharge.^55^ A smaller Δ*E*_p_ is related to a better reversible performance during the redox reaction. In this regard, the Δ*E*_p_ was reduced for the TNO15-800-C-700 and TNO15-800-C-Cu-700 compared to those of the uncoated electrodes. The CV performance of the TNO15-Air-800, TNO15-Ar-800, TNO15-800-C-700, and TNO15-C-Cu-800 half-cells is provided in the Supporting Information Figure S12. To study the redox reaction of the Cu during the charge/discharge, the CV performance of the prepared glucose-CuCl_2_-700 electrode was tested at different voltage ranges from 1.1 to 3.5 V (at a sweep rate of 0.1 mV s^–1^), and the performance is shown in the Supporting Information Figure S13. During the anodic scan, a strong peak observed at 2.7 V is attributed to the oxidation of Cu into Cu_2_O and CuO.^56^

To describe the charge storage kinetics, we evaluated the capacitive and diffusion current contributions to the total capacity using the power law (eq 1),^57^
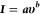
1where *a* and *b* are adjustable parameters, and the *b* value provides insight into the charge storage mechanism. The *b* value would be 0.5 for the semi-infinite linear diffusion and 1 for the surface-controlled process. The fitted log(*i*_*pa*_) vs log(υ) curve for the TNO15-800-C-Cu-700 electrode is shown in Figure 7(b). Obtained *b* values of 0.83 and 0.79 for anodic and cathode peak current density, respectively, show the dominant capacitive contribution to the diffusion-controlled process. Here, the capacitive charge storage mechanism is not limited by solid-state diffusion, which enables fast Li^+^ transportation and results in high-rate capability even at high sweep rates.^57^ This will be further quantitatively analyzed by separating the current response at a particular potential (*i*(*V*)) into capacitive- and surface-controlled *k*_1_υ and Faradaic diffusion-controlled *k*_2_υ^1/2^. Then, the total current at a given potential is represented by eq 2,
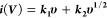
2

Here, υ is the sweep rate and *k*_1_ and *k*_2_ are the slope and intercept of *i*(υ)/υ^1/2^ and υ^1/2^ fitted curves, respectively. The capacitive element and diffusion contribution for the total current at various sweep rates for TNO15-800-C-Cu-700 are calculated and presented in Figure 7(c). The rose-shaded region shows the Faradaic insertion or diffusion element, and the remaining region is the capacitive element. At a sweep rate of 0.2 mV s^–1^, the capacitive contribution is 46.6%, and with an increase in the sweep rate, the capacitive contribution is dominant over the diffusion element. This decrease in the diffusion element is probably due to the lower accessibility of insertion sites at higher sweep rates.

To investigate the Li^+^ transport kinetics and the related Li-diffusion coefficient (coefficient that determines the ionic and electronic transport within the active material) at various discharge–charge states, the galvanostatic intermittent titration technique (GITT) was used. During the measurements, the electrode is subjected to a constant current flux for a short time interval τ, followed by a long relaxation period, and the corresponding potential change is measured as a function of time between the working and the reference electrode (shown in Figure 7(d)), and the process is repeated cyclically. After the constant current pulse, the electrode comes to a nonequilibrium state with a potential called closed circuit voltage (*V_cc_*). During the relaxation process, the Li^+^ ions diffuse from the electrode surface until they reach an equilibrium state and the corresponding *V_oc_* (open circuit voltage). Here, the voltage during the relaxation process is measured as a function of the diffusive process, and the *D_Li_* can be derived.^18^ Hence, the chemical diffusion coefficient of the electroactive species Li^+^ in the prepared TNO15 and the modified samples is estimated through GITT and Fick’s law using the eq 3,^58^
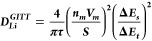
3

Here, τ is the time duration where the constant current is applied (s), *n_m_* is the number of moles of the electrode (mol), *V_m_* is the molar volume of the electrode (cm^3^ mol^–1^), *S* is the electrode/electrolyte contact area (cm^2^), Δ*E*_*s*_ is the steady-state voltage change due to the current pulse, and Δ*E*_*t*_ is the voltage change during the constant current pulse after eliminating the IR drop. The GITT experimental result for the TNO15-800-C-Cu-700 electrode is shown in Figure 7(d). The schematic representation of the current vs time GITT graph is shown in the inset of Figure 7(d), and the scheme of a single GITT measurement is shown in Figure 7(e). The obtained chemical lithium diffusion coefficient (*D_Li_*) as a function of potential for the prepared electrodes during the delithiation is plotted in Figure 7(f). The diffusion coefficient is relatively constant at 2.1 V, and divergent behavior is observed at a potential of 1.7 V, indicating that the material is undergoing a phase change, called the two-phase region (consistent with the plateau region in the charge/discharge curve shown in Figure 5(a)). The diffusion coefficient derived from the intermittent titration technique (both galvanostatic and potentiostatic) based on Fick’s law of diffusion is not a reliable technique for the two-phase region of a phase-changing material. In the two-phase region, ion transport is related to the movement of charges through the interphase boundary and diffusion. Thus, rather than the absolute value, the phenomena are more considerable in the phase transition region. More details can be found in the work by Zhu et al.^59^ The magnitude of *D_Li_* for the TNO15-Ar-800 and the carbon and carbon/coppercoated electrodes is lowered by an order compared to the sample calcinated at 800 °C in air. This points out that the Li^+^ diffusion energy barrier decreases with the introduction of oxygen vacancies and carbon/copper coatings, which facilitate faster lithium-ion transportation.

**Figure 8:**
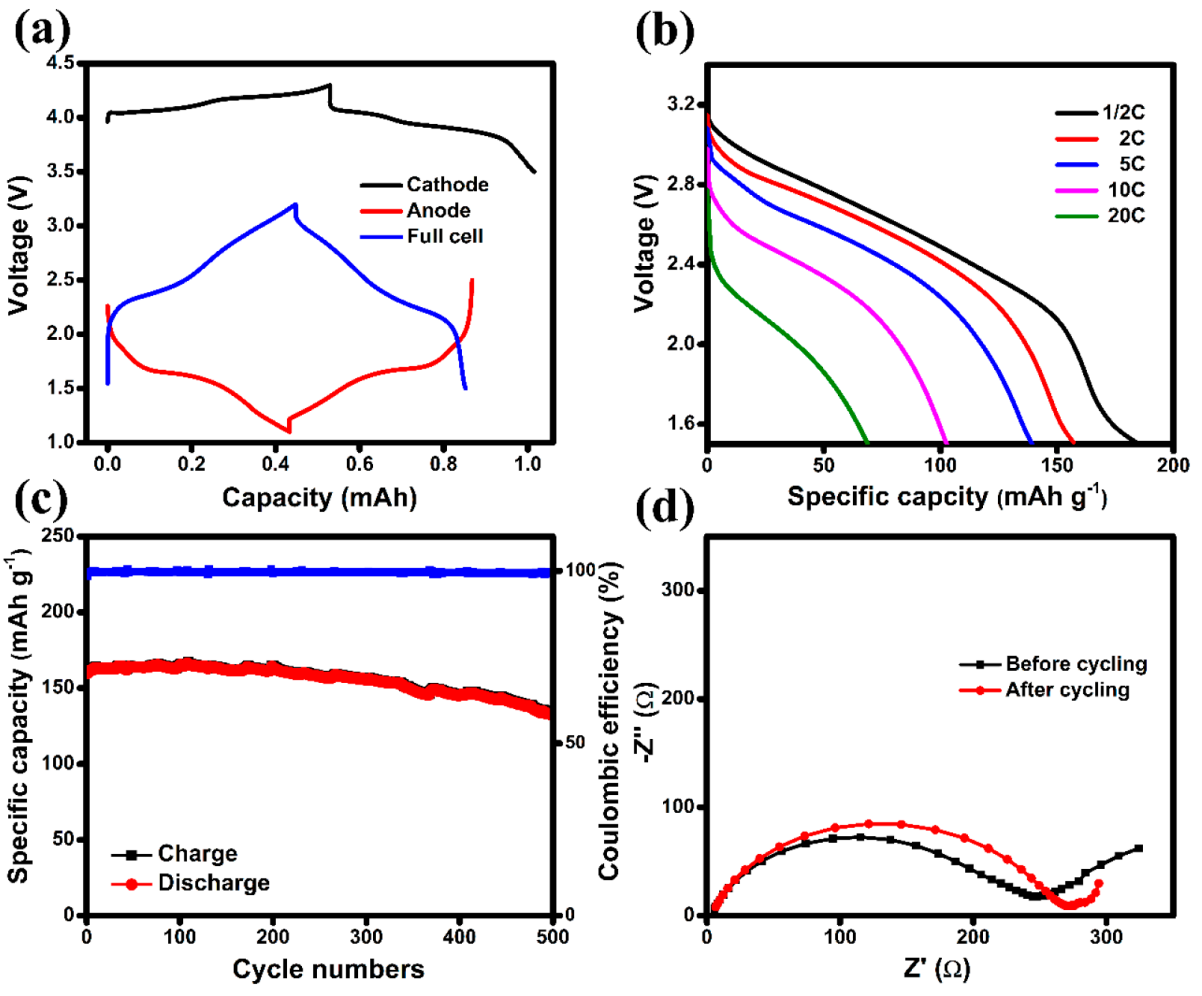
LMO//TNO15-800-C-Cu-700 full-cell electrochemical measurements. (a) The charge/discharge curve of the TNO15-800-C-Cu-700//Li anode (red curve), LMO//Li cathode (black curve), and TNO15-800-C-Cu-700//LMO full-cell (blue curve), (b) discharge curve of the full-cell at different C-rates, (c) cycle stability and Coulombic efficiency result in the full-cell at a charging rate of 2C and discharge rate of 1C for 500 cycles, and (d) EIS performance of the full-cell at 3 V before and after 500 cycles.

To evaluate the potential application of the prepared electrode material, we fabricated a full-cell using commercially available LiMn_2_O_4_ as the cathode and TNO15-800-C-Cu-700 as the anode. LMO is a promising cobalt-free cathode material that provides a high operating voltage (∼4.2 V) to compensate for the drawback of the high operating voltage and high cost of TNO15. Here the charge/discharge window of the full-cell is set to 1.5 to 3.2 V according to the charge/discharge cutoff potential of TNO15-800-C-Cu-700 and LMO shown in Figure 8(a). The N/P ratio is significantly important to the cycle life of the full-cells. Unlike lithium-ion batteries using graphite-based anodes, which are commonly designed as cathode-limited cells, preventing lithium plating, high-power batteries using high-operated voltage anodes such as LTO, TNO12, and TNO15 are often designed as anode-limited cells to achieve a long cycle life. It is because, compared to these anode materials, cathode materials have a fast capacity fading during cycling and an increase in C-rate. Another reason is that these anode materials often have a linear-like charge/discharge curve with a short potential platform. Their anode-limited full-cells can easily cause voltage drift in full-cells, leading to a decrease in capacity.^60^ Therefore, our full-cell, LMO//TNO15-800-C-Cu-700, is anode-limited with an N/P ratio of 0.9. The charge/discharge performance and the cycle stability of the full-cell are tested in constant current mode, and the results are shown in Figure 8(b,c), respectively. As shown in Figure 8(b), the prepared full-cell delivers a high capacity of 186 mAh g^–1^ (based on the mass of TNO15-800-C-Cu-700) at 0.5 C and discharges 159.0, 139.4, 103, and 68.78 mAh g^–1^ capacity at 2, 5, 10, and 20 C, respectively, showing a high-rate discharge capability. However, alternative cathode materials should be selected in future work with better rate capability than raw LMO. The cycle performance of the prepared full-cell was tested at a charging rate of 2C and discharged at 1C for 500 cycles, and the result is shown in Figure 8(c). It shows outstanding stability of capacity in the initial 200 cycles and then decreases slowly in the later 300 cycles. It could be because the capacity of LMO slowly decreased during the 200 cycles, and eventually, the cell became cathode-limited, which accelerated the fading. Overall, the cell still maintains 82.6% capacity even after 500 charge/discharge cycles with 99.4% Coulombic efficiency. The manganese dissolution and low-rate capability of LMO are the limitation factors of the LMO/TNO15 Li-ion batteries. The full-cell impedance at 3 V before and after 500 charge/discharge cycles is shown in Figure 8(d). There is no change observed in the ohmic resistance before and after cycling. However, the diameter of the semicircle in the high- to medium-frequency region is slightly increased because of the increase in charge-transfer resistance after the cycling. This increase in the impedance mainly arises from the structural change on the cathode side and a slight volume expansion over continuous charge/discharge cycles on the anode side.

## Conclusion

In summary, the TNO15-800-C-Cu-700 microsphere, composed of interconnected TNO15 nanoparticles, was synthesized through the solvothermal method followed by calcination in air and argon. The prepared TNO15 electrode has an average lithium storage potential of 1.65 V vs Li/Li^+^, which is sufficient to mitigate the formation of SEI. The LMO//TNO15-800-C-Cu-700 full-cell provides a reversible capacity of 186 mAh g^–1^ at 0.5 C and maintains good rate capability. The thin carbon coating on the surface of the TNO15 nanoparticles with Cu metal increases the ionic and electronic conductivities and reduces the lithium migration barrier. In addition to this, the oxygen deficiencies created inside the samples through calcination in the argon atmosphere boost the ionic conductivity and create additional reaction sites for a better electrochemical performance. From the electrochemical studies, it is evident that the prepared TNO15-800-C-Cu-700 electrode has faster ion diffusion kinetics and higher specific capacity than the other prepared electrodes. From our studies, we also found that the electrochemical performance of the TNO15-C-Cu-800 electrode is comparable enough to TNO15-800-C-700 and TNO15-800-C-Cu-700, which were prepared through a two-step calcination method. Hence, for large-scale and energy-efficient production of this anode, the preferable method could be the single-step synthesis at 800 °C.

## Supporting Information Available

Photographs of the TNO15-Air-800, TNO15-Ar-800, and TNO15-C-Cu-800 samples; additional characterization information including, XRD, BET, XPS, SEM, TEM; calculated specific surface area, pore size, and pore volume; electrical conductivity measurements; Ti^3+^/Ti^4+^ and Nb^4+^/Nb^5+^ peak area ratios and additional electrochemical studies, charge/discharge, in situ XRD, cyclic voltammetry (PDF)
