# Supplementary material for: Insight into the Li-Storage Property of Surface-Modified Ti2Nb10O29 Anode Material for High-Rate Application
Source: ACS Appl Mater Interfaces. 2023 Nov 16;15(47):54568–81. doi: 10.1021/acsami.3c14174 (PMC10694814; doi:10.1021/acsami.3c14174)
Supplement: Supplementary file 1 — am3c14174_si_001.pdf [file am3c14174_si_001.pdf]

Supporting Information

Insight into the Li-Storage Property of Surface-Modified $Ti_2Nb_{10}O_{29}$ Anode Material for High-Rate Application

*Nikhitha Joseph[1], Haojie Fei[1] *, Constantin Bubulinca[1], Marek Jurca[1], Matej Micusik[2], Maria Omastova[2], Petr Saha[3]*

1 Centre of polymer systems, Tomas Bata University in Zlin, 760 01 Zlin, Czech Republic

2 Polymer Institute, Slovak Academy of Sciences, Dúbravská cesta 9, 845 41 Bratislava, Slovakia

3 University Institute, Tomas Bata University in Zlín, 760 01 Zlín, Czech Republic

*Corresponding author e-mail: haojie@utb.cz

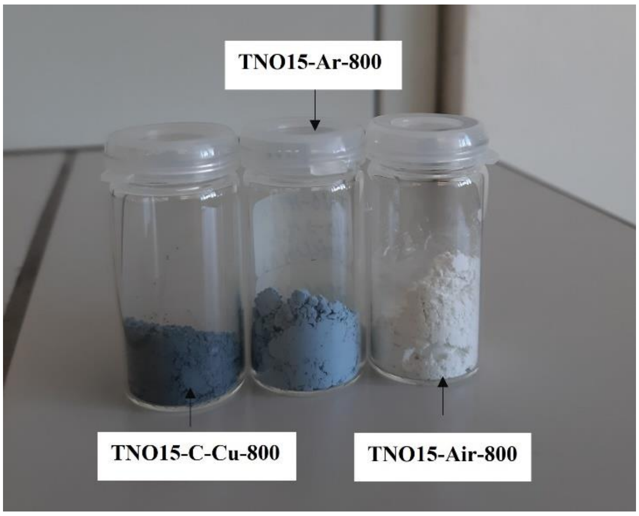

Figure S1. Photograph of TNO15-Air-800, TNO15-Ar-800 and TNO15-C-Cu-800 samples.

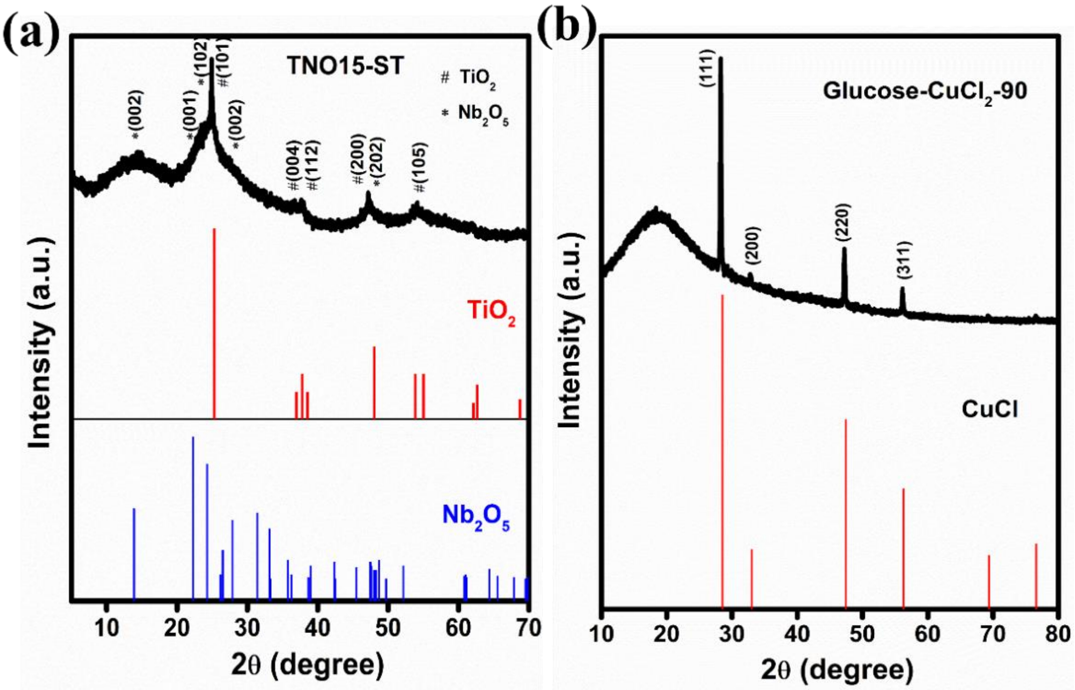

Figure S2. XRD pattern of (a) TNO15 precursor prepared through solvothermal synthesis, (b) Glucose-$CuCl_2$-90 before calcination at 700 °C.

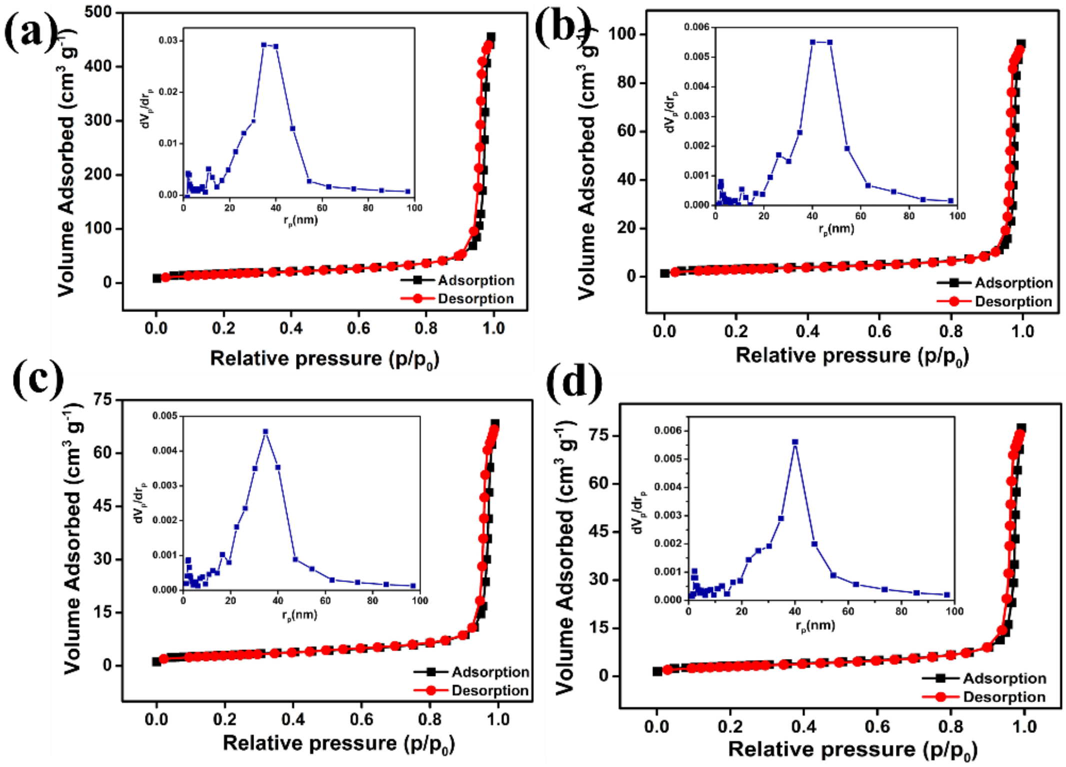

Figure S3. BET and BJH (inset) plot for (a) TNO15-Air-800, (b) TNO15-Ar-800, (c) TNO15-C-Cu-800 and TNO15-800-C-700 samples.

Table S1. BET and BJH data plot for the prepared samples.

Sample code	BET Surface area (m^2 g^{-1})	Pore size (nm)	Pore volume (cm^3 g^{-1})
TNO15-Air-800	63.09	43.72	0.68
TNO15-Ar-800	11.8	48.20	0.14
TNO15-800-C-700	10.8	38.83	0.10
TNO15-C-Cu-800	11.7	39.5	0.11
TNO15-800-C-Cu-700	12.86	33.43	0.10

Table S2. Electrical conductivity measurement of the prepared samples at room temperature.

**Common parameters**	*Material weight: 0.2 g*<br>*Pellet thickness: ~0.62 mm*		*Applied pressure: 6 ton*<br>*Pellet diameter: 13 mm*		
**Sample code**	TNO15-Air-800	TNO15-Ar-800	TNO15-800-C-700	TNO15-C-Cu-800	TNO15-800-C-Cu-700
**Conductivity (S/cm)**	5.6 x 10^{-7}	9.1 x 10^{-7}	4.5 x 10^{-3}	3.1 x 10^{-5}	5.2 x 10^{-3}

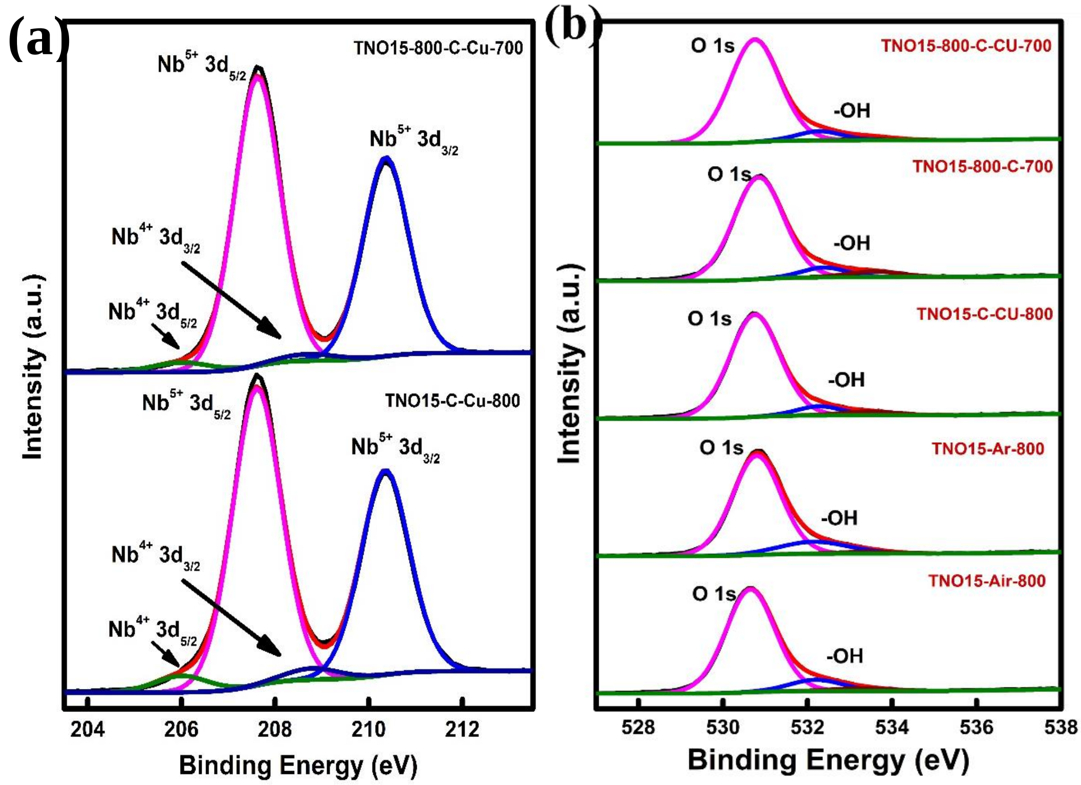

Figure S4. XPS spectra of (a) Nb^{5+} state of TNO15-C-Cu-800 and TNO15-800-C-Cu-700 samples. (b) O 1s spectrum for all the prepared samples.

Table S3. Ti^{3+}/Ti^{4+} and Nb^{4+}/Nb^{5+} peak area ratio in the prepared samples.

Sample code	Ti^{3+}/Ti^{4+}	Nb^{4+}/Nb^{5+}
TNO15-Air-800	0.117	0.0437
TNO15-Ar-800	0.109	0.0497
TNO15-800-C-700	0.115	0.0419
TNO15-C-Cu-800	0.182	0.0613
TNO15-800-C-Cu-700	0.1213	0.0418

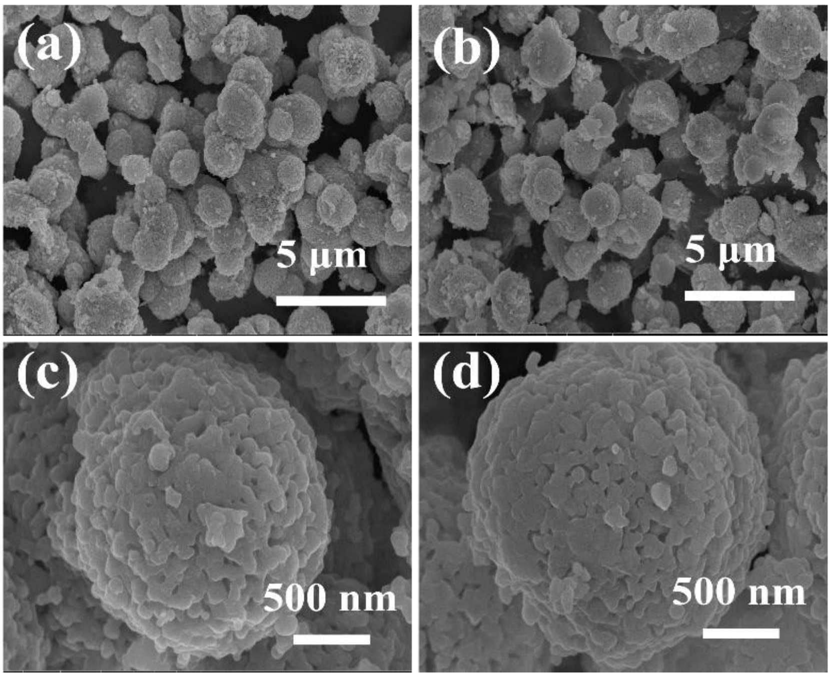

Figure S5. SEM images of (a) & (c) TNO15-Ar-800, (b) & (d) TNO15-C-Cu-800.

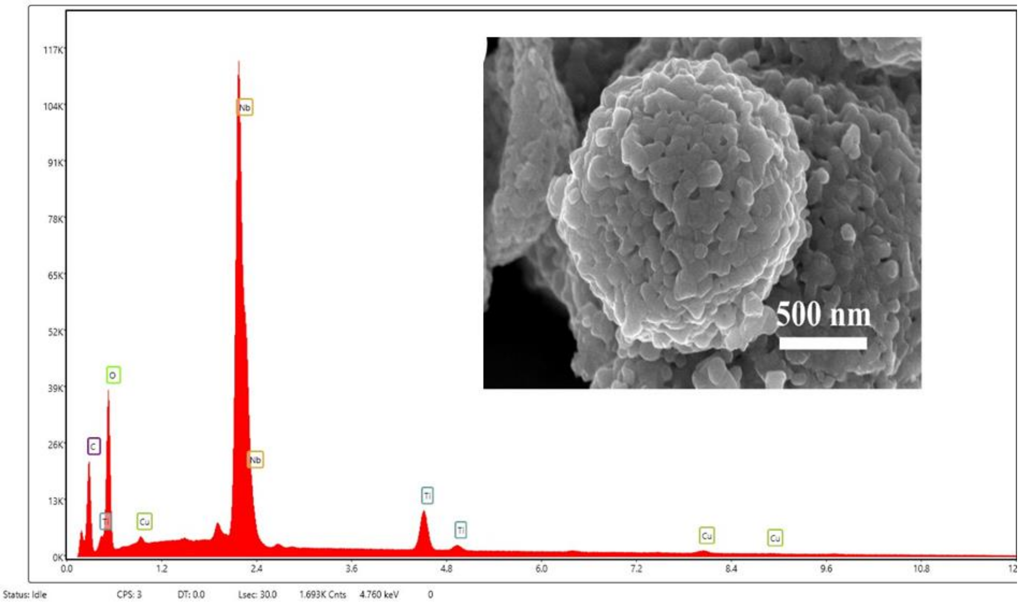

Figure S6. SEM-EDX spectroscopy analysis for the TNO15-800-C-Cu-700 sample.

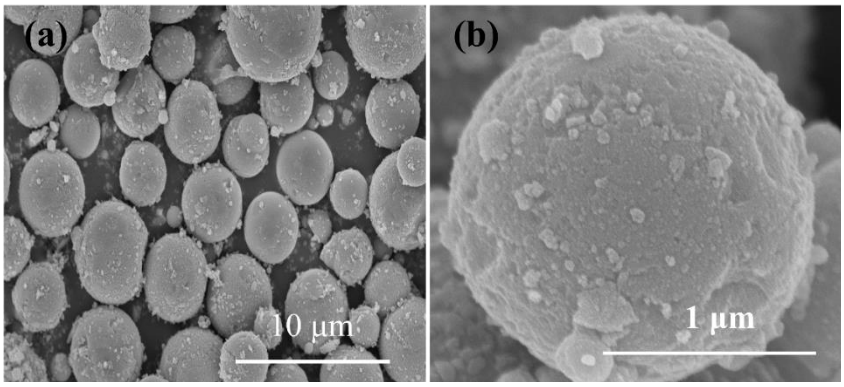

Figure S7. (a) Low and (b) high magnification SEM images of TNO15 precursor prepared through solvothermal synthesis.

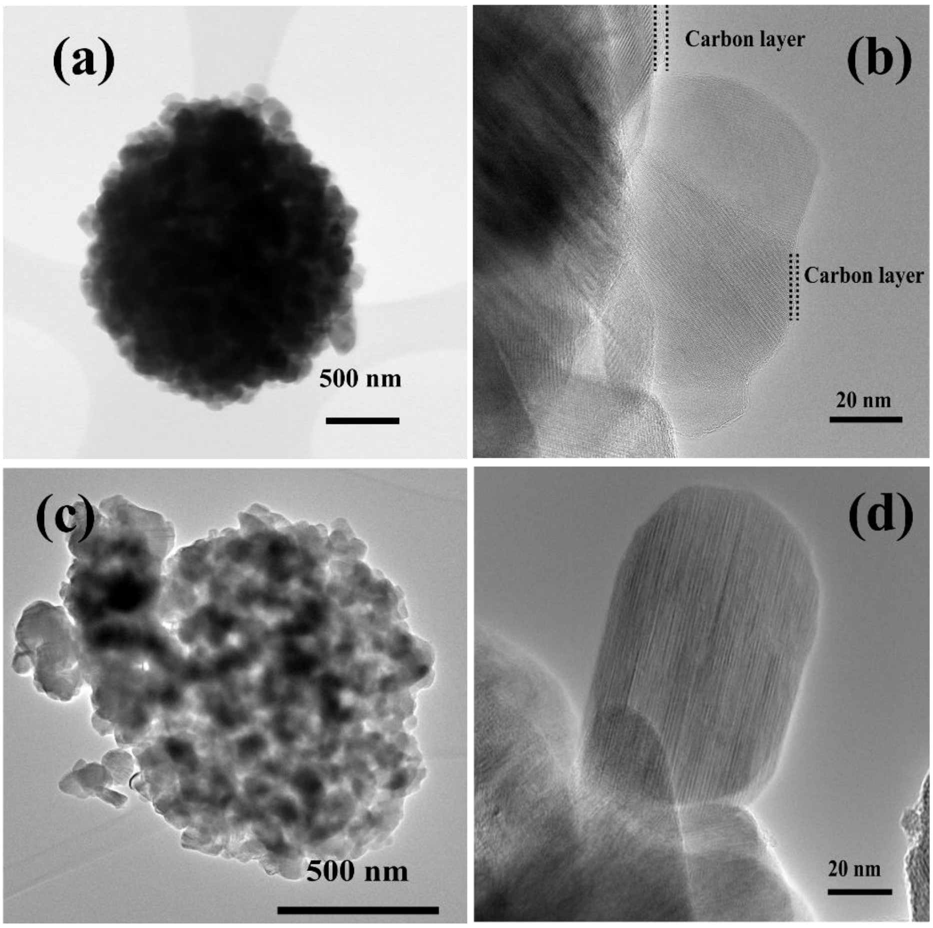

Figure S8. TEM and HRTEM images of (a) & (b) TNO15-800-C-700 and (c) & (d) TNO15-C-Cu-800 samples.

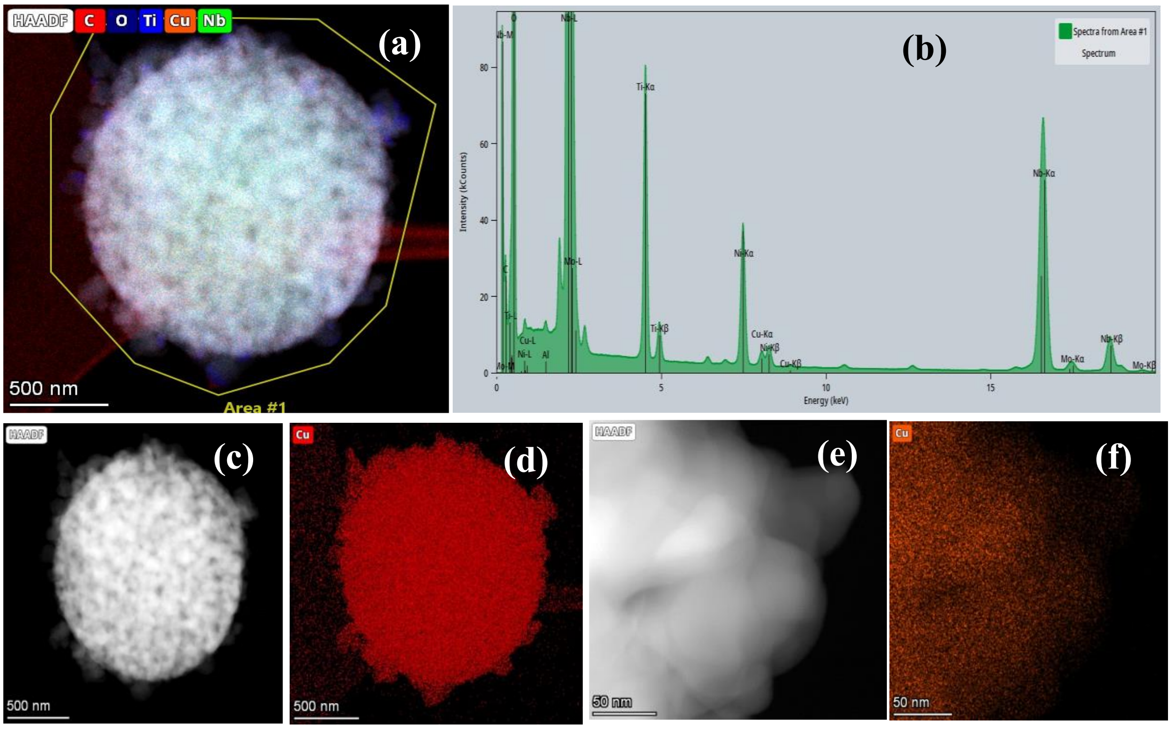

Figure S9. STEM-HAADF elemental mapping from different magnifications for the TNO15-800-C-Cu-700 sample to confirm the presence of Cu on the surface of TNO15.

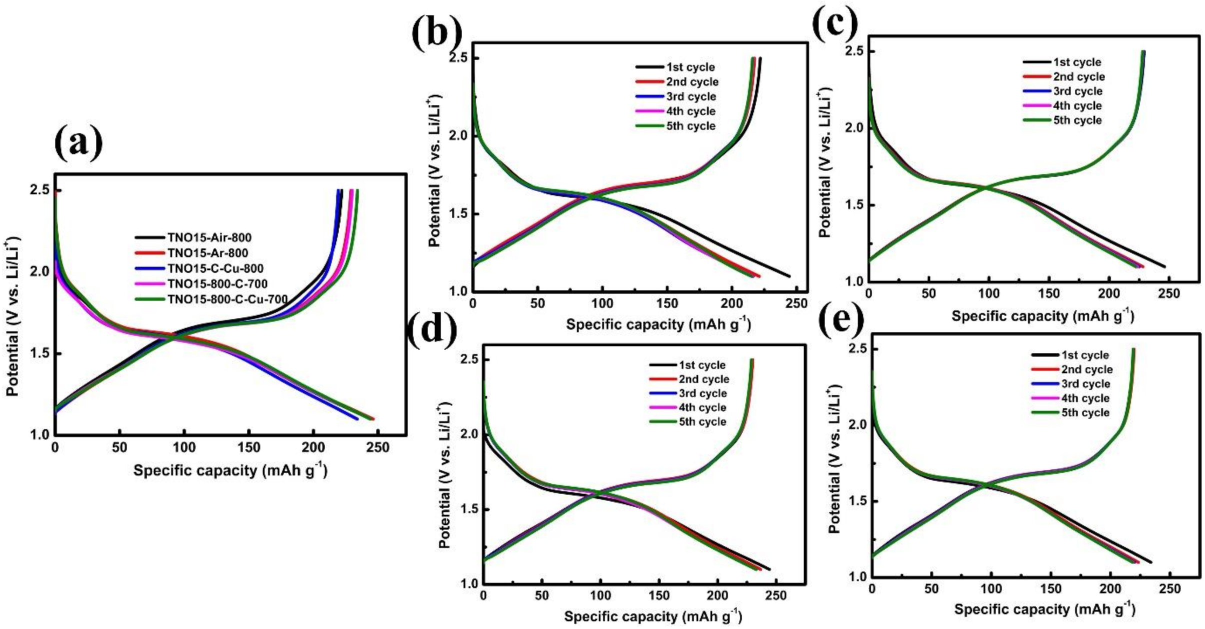

Figure S10. (a) Comparative CD performance of the prepared electrodes at 0.05 A g^{-1}. First 5 CD cycles for (b) TNO15-Air-800, (c) TNO15-Ar-800, (d) TNO15-800-C-700 and (e) TNO15-C-Cu-700 electrode at 0.05 A g^{-1} current density.

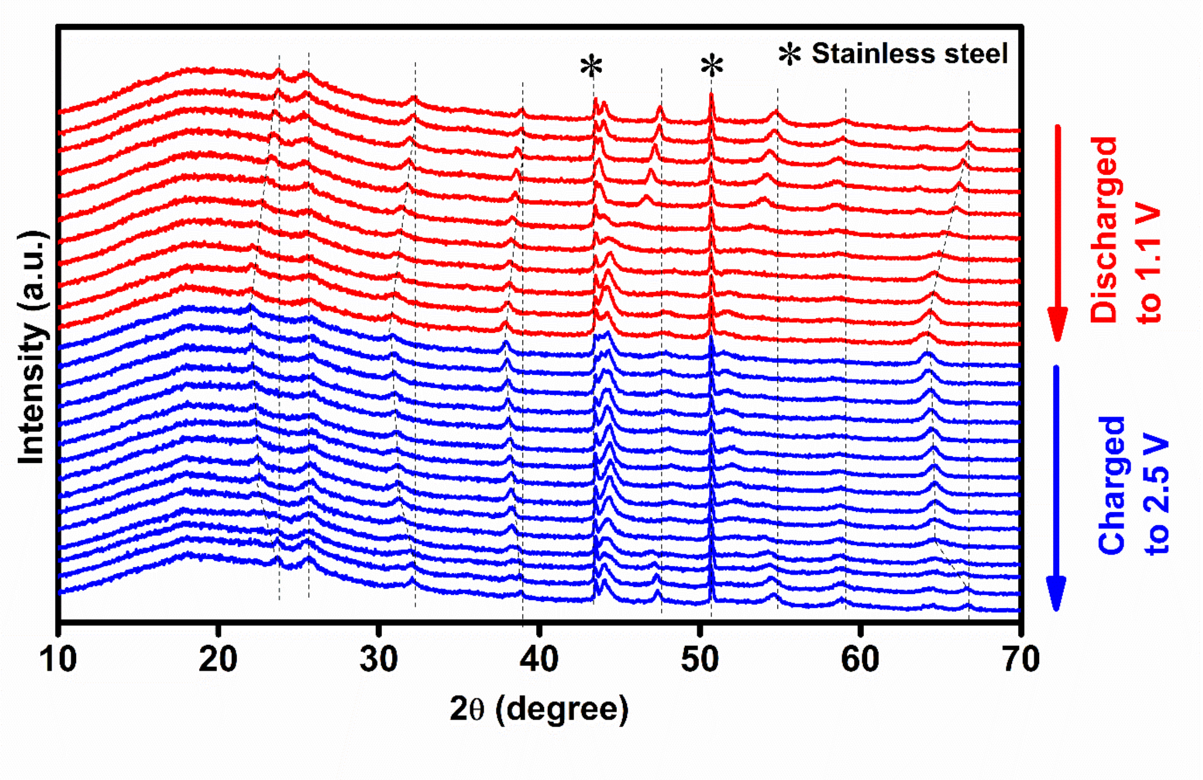

Figure S11. In situ XRD patterns for TNO15-800-C-Cu-700 electrode at 0.1 A g^{-1} current density.

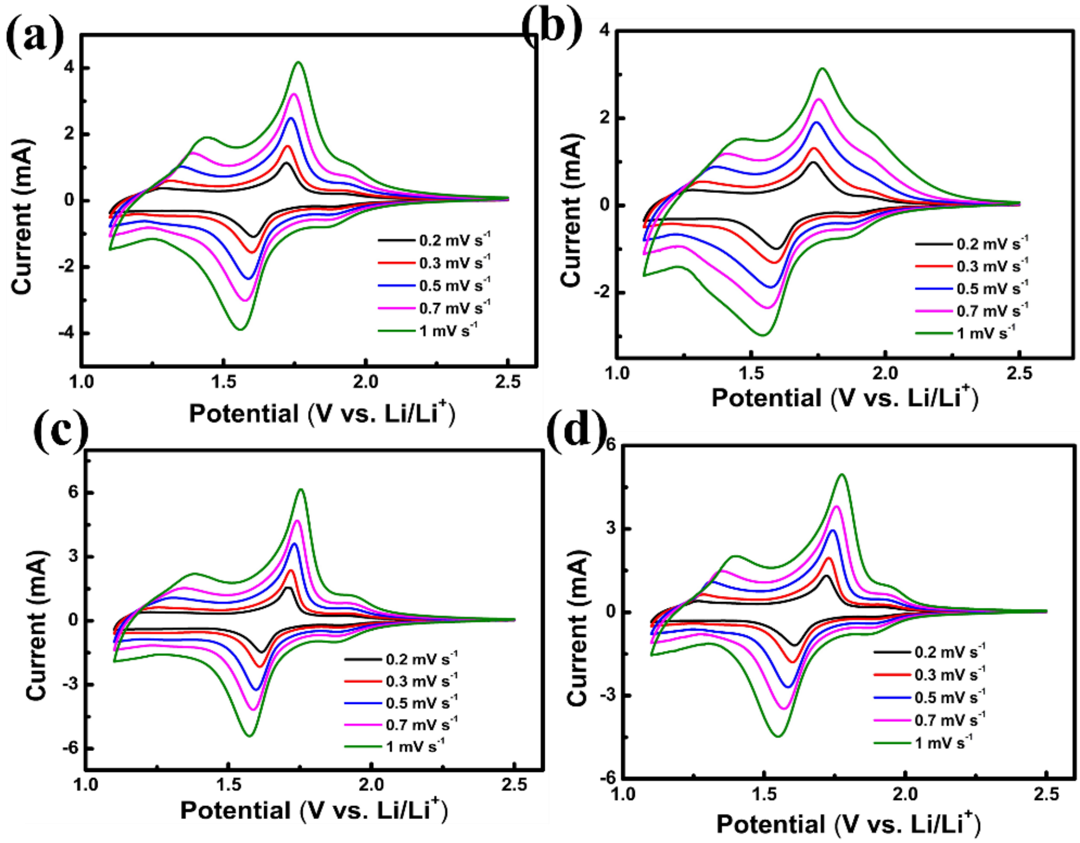

Figure S12. CV performance of (a) TNO15-Air-800, (b) TNO15-Ar-800, (c) TNO15-C-Cu-800, and (d) TNO15-800-C-700 electrodes.

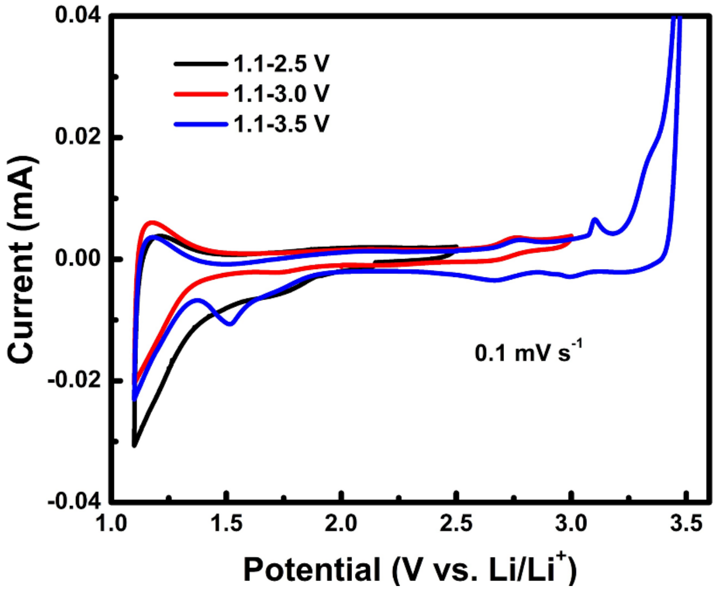

Figure S13. CV performance of Glucose-$CuCl_2$-700 electrode at 0.1 mV s^{-1} for different potential ranges.
